# Supplementary material for: Cooperation in the face of thresholds, risk, and uncertainty: Experimental evidence in fisher communities from Colombia
Source: PLoS One. 2020 Dec 28;15(12):e0242363. doi: 10.1371/journal.pone.0242363 (PMC7769437; doi:10.1371/journal.pone.0242363)
Supplement: S1 File — (PDF) [file pone.0242363.s001.pdf]

Cooperation in the face of thresholds, risk, and uncertainty

S1 File

Appendix 1. Game instructions

Normal text: read out loud to the participants
<Italics: Actions to take by experimenters and assistants>
"——" means: take a short break while talking
Something underlined needs to be emphasized

Welcome and thank you for coming and participating in this activity! You have been invited to participate in this activity as part of a study about natural resource management. In this study, the participation of people like you is very important, because of your work and daily experience managing fishing resources. We are a group of researcher from the Beijer Institute of Ecological Economics and Magdalena University. My name is Juan Carlos Rocha and all members of our team have their name written on their nametag. Thanks to your participation we all can learn from this study. During this activity, we will play a decisions game. And after the game, we will interview each of you. In total, the whole activity will take approximately 3 hours of your time.

As announced earlier, you will receive a minimum of $\$15.000$ pesos by taking part in this activity. This means, no matter the result of the game, you will receive $\$15.000$ Pesos. Depending on the decisions you make in the game, you can earn extra money. You will receive the money at the end of the activity – after you have been interviewed – and paid in private.

You might ask: Why do we use money? – We do NOT see the money you earn as a payment for taking part in this activity, nor the reason for you to be here. — We use money because the exercise requires that you make decisions that have economic consequences. It is to make the game realistic. Please note that if you have participated in any other economic game in the past, the game we will play today is completely different.

Before we start, we would like you to sign a consent form. – The consent form is a payment authorization that states that you are here voluntarily. Next, I will read the consent form out loud in case anyone forgot his/her glasses or have difficulties reading. <read consent form>. In summary, the consent form states that you are here voluntarily. Please note that the consent form is also an agreement between you and us that you will participate in the whole activity. If you or one of the members of your group leaves before the end, neither you or your group members can finalize the activity and – therefore – won't receive any money. Please sing up the consent form now and the assistants will pass collecting them.

Explain common access to a fishing ground

In this game, we want you to imagine that you – as a group – have common access to a fishing ground.

<Point to the bowl on the table in front of them with something in that represents fish>

Although in reality it is impossible to know exactly how much fish there is in a given fishing ground – in this game, we ask you to pretend that we can know exactly how much fish there is. We also assume that there is only one fish species in the fishing ground. And each of you can catch this fish from this common fishing ground.

Explaining decision-making

The game has two stages, each stage will last several rounds, and in each round you will take an individual decision of how much fish to catch. We will take care that the other members of your group wont see your decisions. This means that you can talk about whatever you want, even about how much you want to fish.

<Introduce and show the decision cards. Explain what to write where on the card. Show them the decision cards and explain: >

Each round in the game is equivalent to a day of fishing. On the left column "Individual fish catch" <point it out> you indicate how much fish you want to catch. Our assistants are here to help you if you need it, in case you have difficulties writing, reading, or if you have any questions about the game. In each round you can chose the amount of fish available or not fish at all, which is zero. Bear in mind that your decision affect your individual benefits as well as the benefits of the community.

For each fish you catch, you get 500 Pesos. So – for example – if you catch 10 fish, you will earn 10 * 500 = 5.000 Pesos. (Additional to the 15.000 Pesos you already earned by participating in this activity). You can track your individual earnings on the second column of your decision card coloured in gray *<point it out>*. We will give you calculators and if you need any help please do not hesitate on contact any of our assistants to help you out with the accounting.

Once you have made your decision, we will collect your decision cards at the end of each round. – Please close your decision card when you hand it out to the assistant. We will help you to keep the privacy of your decisions and your group partners.

Once we have collected your decision cards, we will calculate how much fish there will be available for your group in the next round and then inform you about it by telling you and by writing it here *<point it out>* on the board.

Apart from your decisions of you and your group members on how much to fish in each round, the fish stock is also affected by reproduction.

Explain that the fish stock replenishes depending on the stock size

Now I will explain how the fish stock reproduces, using the illustration here <textitpoint to illustration with the fish> . The fish stock grows (or not) between each round of the game. How much the fish stock grows depends on how much fish there is – it is the population size or fish bank. We start with 50 fish in the first round. And you see here that…

<Walk them though the illustration.>

– If there are 46-50 fish, there is no fish population growth. The fish stock available is in that case in fact the same in the next round. *<If participants ask why, answer: there is too high competition for food. They are too many in relation to the size of the fishing ground.>*

– If there is 35-45 fish, there will be 5 more fish in the next round. *<If participants ask why, answer: there is some competition for food, as they are so many in relation to the size of the fishing ground. This results in that the fish stock does not grow as much as between 24 and 34>*

– If there is 20-34 fish, there will be 10 more fish in the next round. *<If participants ask why, answer: There is enough fish so that they can find mating partners and not too much fish so they do not have to compete for food and space>*

– If there is 5-19 fish, there will be 5 more fish in the next round. *<If participants ask why, answer: There is very little fish so to find mating partners is difficult>*

– If there is less than 5 fish, the fish stock doesn't grow either (as for stock size between 46-50). *<If participants ask why, answer: there is too little fish so they cannot find mating partners>*

As I said before, this game lasts several rounds and has two stages. We will tell you when the first stage finishes and explain what happen on the second stage. – We will not tell you the exact number of rounds that you will play in each stage. However, you know that we will play a maximum of 2 hours. – Remember: if there is no more fish, the game stage ends and you will not earn any more money. As far as there is fish stock to fish, the game stage continues and you will be able to make some money. Also remember: your decisions affect your individual earnings but also affect the benefits of the group with whom you share the fish stock.

Do you have any questions so far before we go through an example?

<If someone asks about how to share a harvest that is larger than the stock, answer: we will share proportionally according to your catch claim>

Examples

<Use the bowl on the table for it, i.e., take out and put in fish accordingly when describing the example. Also actively engage the participants in the example, i.e., ask them about what they think the reproduction is in the given examples>

There are 50 fish in the beginning of the game. Now - let's say - you catch together in the first round 12 fish (for example: you take 4, you 3, you also take 3 and you 2). *<take 12 fish out of the bowl>* So now there are then 38 fish remaining, that is 50 fish minus 12. Now we can check here on the board *<point to illustration>* the reproduction of a fish stock with size 38. How much do you think fish will reproduce? *<ask the participants>* - Yes! Exactly, it is 5 more. *<add the five fish>* So in the next round the fish stock will be then 43 fish (50 – 12 + 5) and this number we will then communicate to you out loud and write up here *<point to where the stock size is displayed for each round>*.

Let's do another example… So now there is 43 fish in the bowl *<Ask a fishermen to be the assistant>*. If you then catch 13 fish in total *<take out 13 fish>*, there are then 30 fish left and the stock will then grow with how much more fish for next round? *<ask participants>* Exactly! 10 fish more. *<add the 10 fish>* so then at the beginning of the next round you would start with 40 fish.

If your group catch is in any round bigger than the available catch, the game stage ends, as there is no more fish to catch. In that case, the share of fish will be divided proportionally.

<Example if they ask: For example, if there is only 10 fish on the fishing ground but your collective catch is 100 fish (let's say 30, 20, 30, 20), the remaining 10 fishes will be divided proportionally to your intended catch. So if you intended to take 30 out of 100 as a group (30%), then you get 30% of the actually fish available, this is 3>

– So now you know how to indicate your catch decisions on your decision card in each round or fishing day – and you also know how to calculate the size of the fish stock for the next round: you subtract the sum of your individual catch decisions from the current fish stock and then you add reproduction accordingly. Remember, we do the calculations for you but we also want you to understand how it works. Any questions so far?

Now let's get to the third column on the decision card. The first and second column correspond to individual fishing and earnings *<point them out>*. We want you to note down there what you think the size of the fish stock will be in the next round – what you think we will write on the board to start the next round. You decide how much to fish in each round, make your accounting of individual earnings or ask an assistant to help you with the accounting, and also speculate about what will happen with the stock size for next round. Then we also make the accounting and communicate publically what happen with the collective resource but we wont reveal who fished what, only what happen at the level of the group. Thus, you can compare what you expected to happen (the third column) with what it really happens (the public table with the fish stock state).

Communication

… throughout the whole game and from the very beginning, you are allowed to talk to each other. This means that you can talk about whatever you want, about your decisions, about the rules of the game, strategies and even how much you want to fish. You cannot make any threats or arrangements for side-payments during or after this activity. You have two minutes in between rounds to discuss with other members of your group how much you want to fish, after the two minutes you can write your individual decision. We keep the account of time and will inform you when you need to hand out your decision cards.

Do you have any questions? — Please also remember, – in case you have any questions during the game, please don't hesitate on asking any of us. We will clarify your doubts.

Group division

You will now be divided into groups of 4 people.

<Make group division. Ask assistants to distribute the player number tags. Each subject randomly picks a name tag, which tells which group (color assigned) he will be assigned to and which number (1-4) he is in that group. So, for example, if we have three groups playing at the same time we could have something like Blue (1,2,3,4); Green (1,2,3,4) and Yellow (1,2,3,4). Keep in mind that people from the same household (e.g., siblings, cousins) or close friends should participate in different groups if possible>

Now that the groups are formed, could you please raise your hand if any member of your household or close friend is on the same group as you?

<From now on each group works with their own experimental leader. Each leader summarizes the rules of the game before starting the practice rounds>

On a different room:

Good morning / afternoon. My name is *<your name here>* and our assistant is *<assistant name>* and we will guide you throughout the game. Now you are a group of 4 fishermen who share the same fishing ground with 50 fish. In front of you, you will find a folder with your decision cards, calculators and a pen. If you need help writing or keeping the count, please let us know, our assistant and me are here to help you.

Practice rounds

Before we start the first stage of the game we are going to do some practice rounds to make sure we all have understood the instructions. Please note: you do not earn any money during the practice rounds. During the practice rounds we do not reveal who took what, only what "someone took" and we will do the calculation of the stock size openly together so that everyone can follow it. Now you have two minutes that you can use to discuss your decisions, after that time you must write your decision and we will gather the decision folders.

<We pick up the decision cards and bring them to Caroline. She will return a paper with the individual catch on a random order so you can read it publically. Use the bowl with the fake fish on the table, i.e., take out fish and then add fish to the bowl accordingly>

Okay, during this practice some one caught *<take out fish from the bowl as you read>*, another person caught…, the third person… and the last one…. *<make sure to count how many fish is left in the bowl afterwards with the fishermen>*. Therefore, the fish stock is left with XX fish. So, how many fish will reproduce for the next round?

<Let them answer. Once they had calculated the new stock size write it down on the poster in front of round P1 (practice round 1). Repeat the practice round 2 or 3 times and make sure everyone has understood the dynamics of the game>

Any questions?

If not, let's start the first stage of the game. – This means that from now on you will earn some money according to the decisions you make In case you listen to other groups playing, you should know that what happens in other groups is not related to what happen in our group game. Maybe they finish earlier, but that does not mean that the game you are participating will also end. Before we start the first stage of the game for real, here is a short summary of the rules of the game:

- The four of you share together a fishing ground.
- In each round, you will take an individual and private decision of how many fish to catch. You can choose to fish as much fish as there is available or not fishing at all (zero). For each fish you capture you earn 500 pesos. You will also guess how many fish will be left in the stock for the next round.
- Your decisions affect your individual earnings but also the resource of the community.
- As long as there is fish left, this stage of the game continues and you can keep earning money.
- If the resource collapses, this stage of the game ends.
- We do not tell you how many rounds you will play.
- The fish reproduction depends on how much fish there is left on the fish stock *<point to illustration and remind them of the tables they got>*
- You are not allowed to show each other what you write down on your decision cards.
- Remember, you are allowed to talk in the game. This means you can talk about whatever you want even of how much you want to fish for a maximum of 2 minutes time between rounds. After the 2 minutes, you need to write down your decision.

Do you have any questions?

—- end of practice rounds, start of the game —-

The fish stock starts with 50 fish, now you have two minutes that you could use to discuss your decisions. After that time you need to write your decision and we will pick up the decision folders.

<After each round you announce the new stock size>

The new stock size is […]

<The group assistant adjust the stock size in the bowl accordingly – Bear in mind that you only need to update the stock size, do not reveal the individual catch when you put or add fish tokens>

<Example if they ask: For example, if there is only 10 fish on the fishing ground but your collective catch is 100 fish (let's say 30, 20, 30, 20), the remaining 10 fishes will be divided proportionally to your intended catch. So if you intended to take 30 out of 100 as a group (30%), then you get 30% of the actually fish available, this is 3>

Introducing the treatments

Threshold treatment

<after 6 rounds>

Now we are going to play the second stage of the game. In this round, we want to inform you about the new stock size, which is […*<write down the stock size for next round>*]. We also want to inform you that a climate event – with long lasting and negative consequences for the fishing ground you manage in common might occur.

The climate event will affect the fish reproduction rate, it will decrease below fish stock size of 28 fish. As you can see here *<point out the new illustration with threshold dynamics>*, the reproduction will only be of a fish for stock size between 2 and 27 fish. For any other stock size, the reproduction rate will be the same, between 28-34 it will be 10 fish, between 35-45 it will be 5 fish, as you can see here*<point out>*. The stock size between 0-4 and between 45-50 wont reproduce extra fish.

Thus, the climate event brings an abrupt reduction on the fish growth if the population size is 28 fish or less. *<if participants ask why: the climate event can reduce rain showers, increase water temperature and the risk of droughts. These events impact nutrient availability for fish and the fish reproduction as well as other species in the ecosystem on which fish depends>*

The assistant will hand out a new table showing the relationship between population size and the reproduction after the climate event *<hand out>*. From this round on and to the end of the game the climate event occurs and it affects the resource's reproduction.

With this new information, we will also reestablish the population size back to 50 fish *<here please strikethrough the number of fish left last round of the first stage and clarify that the stock size starts again with 50 fish>*, just as the beginning of the game. All other rules remain the same:

- The four of you share together a fishing ground.
- In each round, you will take an individual and private decision of how many fish to catch. You can choose to fish as much fish as there is available or not fishing at all (zero). For each fish you capture you earn 500 pesos. You will also guess how many fish will be left in the stock for the next round.
- Your decisions affect your individual earnings but also the resource of the community.
- As long as there is fish left, this stage of the game continues and you can keep earning money. If the resource collapses, this stage of the game ends.
- We do not tell you how many rounds you will play.
- The fish reproduction depends on how much fish there is left on the fish stock and if the climate event occurs or not *<point to illustration and remind them of the tables they got>*
- You are not allowed to show each other what you write down on your decision cards.
- Remember, you are allowed to talk in the game. This means you can talk about whatever you want even of how much you want to fish for a maximum of 2 minutes time between rounds. After the 2 minutes, you need to write down your decision.

The only difference is that – a climate event did happen and from now on the resource reproduction is lower if the population size is below 28 fish.

Any questions?

If not, let's keep playing. The fishing ground restarts with 50 fish, now you have 2 minutes that you could use to discuss your decisions. After that time you need to write your decision and we will pick up the decision folders.

Uncertainty treatment

<after 6 rounds>

Now we are going to play the second stage of the game. In this round, we want to inform you about the new stock size, which is […*<write down the stock size for next round>*]. We also want to inform you that a climate event – with long lasting and negative consequences for the fishing ground you manage in common might occur.

These kinds of climate events are highly unpredictable. This means that you do not know whether and when this event will happen. It could happen in the next round, in four rounds, – or never…

In case the event does happen, – the reproduction of fish will reduce below a stock size of 28, – as you can see here *<put up visualization of threshold stock dynamics>* reproduction will be only 1 for stock sizes between 2-27. Reproduction stays the same for stock sizes between 28 and 34 (it will be 10) and between 35 and 45 (it will be 5), as you can see here *<point to it>*. And for stock sizes between 0-4 and 45-50, do not reproduce.

Thus, the climate event brings an abrupt reduction on the fish growth if the population size is 28 fish or less. *<if participants ask why: the climate event can reduce rain showers, increase water temperature and the risk of droughts. These events impact nutrient availability for fish and the fish reproduction as well as other species in the ecosystem on which fish depends>*

The assistant will hand out a new table showing the relationship between population size and the reproduction after the climate event *<hand out>*.

We also do not know whether and when this event might happen. I will now tell you how we will determine in which round the event will happen:

We have here two bowls – one with 5 green and the other one with 5 red *<point out>* balls in it. The red ball represents the climate event, the green one means nothing changes – reproduction stays the same. We will now take 1 white and 1 red balls *<mix in the third non-transparent urn>* and then [*name of person*] will take out 8 of the remaining balls from this bowl, in which we mixed the remaining green and red balls. However, (s)he will do this under the cover of a cloth – so we all won't know the amount of white and red balls among the four balls that (s)he picks. *< hold a cloth over both urns so that everyone can see that (s)he takes out balls and also how many but neither we nor the participants can see the color>*. So now we have here an urn with 10 balls with 1 white, 1 red and 8 balls of which color is uncertain both for you and us.

At the beginning of each new round – before you make your decisions – from now on, [*assistant name*] will draw a ball – but without any of you seeing the color of the ball (s)he draws – If (s)he draws a green ball, nothing will change in that round. But if (s)he will draw the red ball, the climate event will happen. This means we would then calculate the stock size according to the new relation between stock size and reproduction *<point to the table again>*.

As I mentioned before, the event is long lasting. This means, once we drew a red ball, the new relation between stock size and reproduction will apply until the end of the game. Please note, however, that regardless whether we draw a red ball or not, we will continue drawing. Remember – the occurrence of the event is unpredictable and we also do not know whether and when it will happen.

With the introduction of this information, we will also reset the stock size to 50 *<here please strikethrough the number of fish left last round of the first stage and clarify that the stock size starts again with 50 fish>*, like in the beginning of the game. All other rules remain exactly the same:

- The four of you share together a fishing ground.
- In each round, you will take an individual and private decision of how many fish to catch.
- You can choose to fish as much fish as there is available or not fishing at all (zero). For each fish you capture you earn 500 pesos. You will also guess how many fish will be left in the stock for the next round.
- Your decisions affect your individual earnings but also the resource of the community.

- As long as there is fish left, this stage of the game continues and you can keep earning money. If the resource collapses, this stage of the game ends.
- We do not tell you how many rounds you will play.
- The fish reproduction depends on how much fish there is left on the fish stock and if the climate event occurs or not *<point to illustration and remind them of the tables they got>*
- You are not allowed to show each other what you write down on your decision cards.
- Remember, you are allowed to talk in the game. This means you can talk about whatever you want even of how much you want to fish for a maximum of 2 minutes time between rounds. After the 2 minutes, you need to write down your decision.

The only difference is that – a climate event could happen in the following rounds.

Do you have any questions?

If not, let's keep playing. The fishing ground restarts with 50 fish, now you have 2 minutes that you could use to discuss your decisions. After that time you need to write your decision and we will pick up the decision folders.

<Example if they ask: For example, if there is only 10 fish on the fishing ground but your collective catch is 100 fish (let's say 30, 20, 30, 20), the remaining 10 fishes will be divided proportionally to your intended catch. So if you intended to take 30 out of 100 as a group (30%), then you get 30% of the actually fish available, this is 3>

Risk treatment

<after 6 rounds>

Now we are going to play the second stage of the game. In this round, we want to inform you about the new stock size, which is […]*<write down the stock size for next round>*. We also want to inform you that a climate event – with long lasting and negative consequences for the fishing ground you manage in common can occur.

These kinds of climate events are highly unpredictable. This means that you do not know whether and when this event will happen. It could happen in the next round, in four rounds, – or never…

In case the event does happen, – the reproduction of fish will reduce below a stock size of 28 fish, – as you can see here *<put up visualization of threshold stock dynamics>* reproduction will be only 1 for stock sizes between 2-27. Reproduction stays the same for stock sizes between 28 and 34 (it will be 10) and between 35 and 45 (it will be 5), as you can see here . And for stock sizes between 0-4 and 45-50, do not reproduce.

Thus, the climate event brings an abrupt reduction on the fish growth if the population size is 28 fish or less. *<if participants ask why: the climate event can reduce rain showers, increase water temperature and the risk of droughts. These events impact nutrient availability for fish and the fish reproduction as well as other species in the ecosystem on which fish depends>*

The assistant will hand out a new table showing the relationship between population size and the reproduction after the climate event *<hand out>*.

We also do not know whether and when this event might happen. I will now tell you how we will determine in which round the event will happen:

We have here two bowls – one with 5 green and the other one with 5 red *<point out>* balls in it. The red ball represents the climate event, the green one means nothing changes – reproduction stays the same. [*Name of person*] will take the 10 balls (5 green and 5 red) and mix them on the bowl. At the beginning of each new round – before you make your decisions – from now on, [*assistant name*] will draw a ball – but without any of you seeing the color of the ball (s)he

draws – If (s)he draws a green ball, nothing will change in that round. But if (s)he will draw the red ball, the climate event will happen. This means we would then calculate the stock size according to the new relation between stock size and reproduction *<point to the table again>*.

As I mentioned before, the event is long lasting. This means, once we drew a red ball, the new relation between stock size and reproduction will apply until the end of the game. Please note, however, that regardless whether we draw a red ball or not, we will continue drawing. Remember – the occurrence of the event is unpredictable and we also do not know whether and when it will happen. But we both know the probability is 50-50.

With this new information, we will also reset the stock size to 50 *<here please strikethrough the number of fish left last round of the first stage and clarify that the stock size starts again with 50 fish>*, like in the beginning of the game. All other rules remain exactly the same:

- The four of you share together a fishing ground.
- In each round, you will take an individual and private decision of how many fish to catch. You can choose to fish as much fish as there is available or not fishing at all (zero). For each fish you capture you earn 500 pesos. You will also guess how many fish will be left in the stock for the next round.
- Your decisions affect your individual earnings but also the resource of the community.
- As long as there is fish left, this stage of the game continues and you can keep earning money. If the resource collapses, this stage of the game ends.
- We do not tell you how many rounds you will play.
- The fish reproduction depends on how much fish there is left on the fish stock and if the climate event occurs or not *<point to illustration and remind them of the tables they got>*
- You are not allowed to show each other what you write down on your decision cards.
- Remember, you are allowed to talk in the game. This means you can talk about whatever you want even of how much you want to fish for a maximum of 2 minutes time between rounds. After the 2 minutes, you need to write down your decision.

The only difference is that – a climate event could happen in the following rounds.

Do you have any questions?

If not, let's keep playing. The fishing ground restarts with 50 fish, now you have 2 minutes that you could use to discuss your decisions. After that time you need to write your decision and we will pick up the decision folders.

<Example if they ask: For example, if there is only 10 fish on the fishing ground but your collective catch is 100 fish (let's say 30, 20, 30, 20), the remaining 10 fishes will be divided proportionally to your intended catch. So if you intended to take 30 out of 100 as a group (30%), then you get 30% of the actually fish available, this is 3>

Base line

<after 6 rounds>

Now we are going to play the second stage of the game. In this round, we want to inform you about the new stock size, which is […]*<write down the stock size for next round>*. We also want to inform you that this has been a really good year and the fish stock recover more than normal. The stock size is 50 fish *<here please strikethrough the number of fish left last round of the first stage and clarify that the stock size starts again with 50 fish>*, like in the beginning of the game. All other rules remain exactly the same:

- The four of you share together a fishing ground.
- In each round, you will take an individual and private decision of how many fish to catch. You can choose to fish as much fish as there is available or not fishing at all (zero). For each fish you capture you earn 500 pesos. You will also guess how many fish will be left in the stock for the next round.
- Your decisions affect your individual earnings but also the resource of the community.
- As long as there is fish left, this stage of the game continues and you can keep earning money. If the resource collapses, this stage of the game ends.
- We do not tell you how many rounds you will play.
- The fish reproduction depends on how much fish there is left on the fish stock *<point to illustration and remind them of the tables they got>*
- You are not allowed to show each other what you write down on your decision cards.
- Remember, you are allowed to talk in the game. This means you can talk about whatever you want even of how much you want to fish for a maximum of 2 minutes time between rounds. After the 2 minutes, you need to write down your decision.

The only difference is that – a positive event made that the stock size grew more than normal and we start with 50 fish again.

Do you have any questions? If not, let's keep playing. The fishing ground restarts with 50 fish, now you have 2 minutes that you could use to discuss your decisions. After that time you need to write your decision and we will pick up the decision folders.

<Example if they ask: For example, if there is only 10 fish on the fishing ground but your collective catch is 100 fish (let's say 30, 20, 30, 20), the remaining 10 fishes will be divided proportionally to your intended catch. So if you intended to take 30 out of 100 as a group (30%), then you get 30% of the actually fish available, this is 3>

Appendix 2. Questionaire

Information for surveyers:
<Text in italics should not be read outloud, it is recommendations about how to ask or how to guide the fisher in certain questions>
"——" means: take a short break while talking
Something underlined needs to be emphasized

Place:	Date:	Interviewer:	Participant no:
		Group color:	Time:

We remind you that the same as your decisions in the experiment, the information of this survey is completely confidential (besides the researchers, no one else will have access to your answers) and the data is for only for research purposes.

<First introduce yourself and ask the interviewee name to make the exercise more personal>

1. What is your name? ______________________________
2. How satisfied are you with your life?
   Very satisfied (1) Satisfied (2) Disatisfied (3) Very disatisfied (4)

Questions about the game:

Now we will start with some questions related to the game you participated on:

3. Have you participated before in an economic game? Yes __ (1) No __ (0)
4. In the game we did just now - - was someone in your group you usually go out fishing with? Yes __ (1) No __ (0)
5. Were you surprised when the game finished? Yes __ (1) No __ (0)
6. *<If yes>* How many more rounds did you expect that you would played?
   No more rounds __ less than 5 rounds __ more than 5 rounds __

   6.1. Any other comments about the game? *<Capture any relevant experience that the participant wants to share about the game>*______________________________

Description of fishing activities

7. How old were you when you started fishing? __
8. Have you been fishing ever since *<repeat year or age from question above>* (for most of the time)? Yes __ (1) No __ (0)
9. Have you been fishing here *<community name>* ever since (or at least mos of the time) you started fishing? Yes __ (1) No __ (0)
10. *<If not>* Where have you fished before? ____
11. During the last 12 months, were there any months when you did not fish? Yes __ (1) No __ (0)
12. *<If yes>* In whcih months did you <u>not</u> fish?
    Jan__ Feb__ Mar__ Apr__ May__ Jun__ Jul__ Aug__ Sep__ Oct__ Nov__ Dec__
13. What do you do in the months you are not fishing? ________________

In the months you fish…

14. How many days do you go fishing in a normal week? __
15. How many hours do you fish on an average day? __
16. How much fish (in Kg) and how much money (in pesos) do you make at the end of normal, a good and a bad day?

    a) Normal day: __ Kg __ pesos

    b) Good day: __ Kg __ pesos

    c) Bad day: __ Kg __ pesos

17. In the case of a bad day with no catch or earnings at all, how often does that happen?
    Once a year __ Once a month __ Once a week __ Several times a week __

Fishing styles and gears

Now let's talk about how you go fishing…

18. Do you at times fish with other people? Yes __ (1) No __ (0)

    18.1. *<If yes>* How often do you fish with others?
    Rarely __ Half of the times __ Most of the time __ Always __

    18.2. How many is the crew? __

    18.3. Is it always the same crew? Yes __ (1) No __ (0)

    18.4. *<If yes>* Who in the crew decide when to go fishin? Me __ Other person __ [If so, who? ___________]

19. Do you use a boat or a vessel? Yes __ (1) No __ (0)

    19.1. *<If yes>* Are you the captain? Yes __ (1) No __ (0)

    19.2 *<If yes>* Do you own the boat? Yes __ (1) No __ (0)

20. Do you always go to the same fishing place? Yes __ (1) No __ (0)

21. *<If yes>* Where do you go? ________________

22. What are the most important species you fish over the year? How do you capture them? What is the one you fish the most? Which one leaves better profits?

22.1 What are the species you fish the most?	22.2 What arts do you use?	22.3 From what do you fish them?	22.4 From 1-4, which do you fish the most (Kg)	22.5 From 1-4, which leaves better prifts (pesos)

22.6 Is the fishing art that you uses the most own by yourself? Yes __ (1) No __ (0)

Decision-making

How much of your share of the daily catch…

23. … do you consume yourself (your household including what you eat right away and what you keep for storing)
    None __ Some __ Half __ More than half __ All __

24. How much do you sell?
    None __ Some __ Half __ More than half __ All __

    24.1 Who do you sell to? ________________

25. How much do you give away? (e.g. neighbours, friends)
    None __ Some __ Half __ More than half __ All __

Future

26. Do you think you will continue being a fisher, let's say, in the next 10 years?
    Absolutely yes __ Yes __ Absolutely no __ No __ I do not know/no opinion __

27. *<If no>* Why? ________________________________

28. Do you think your children would like to become fishers in the future?
Absolutely yes __ Yes __ Absolutely no __ No __ I do not know/no opinion __

29. *<If no>* Why? ________________________________

Changes in fish abundance, shocks, knowledge and attitudes

30. Have you been landing the same types of species since you started fishing here *<name of place>*? Yes __ No ___

31. *<If no>* How did it change? __________________

32. Is there a certain type of species that you cannot get enough of today in comparison to the past? Yes __ No __

33. *<If yes>* Which ones?______________________________

34. Have you ever experienced a sudden (more dramatic) change in fish abundance? This would be something more dramatic then a seasonal variation, where you really noticed that a particular species seems to have disappeared for a longer time period. Yes __ No __

35. *<If yes>* Please describe how did you noticed that change: what has happened? (which species disappeared, for example), when that was (approx. year) and for how long or ongoing, what you think caused the change:

35.1 What happened? (which fish species)	35.2 When?	35.3 For how long? still ongoing?	35.4 Causes?	35.5 How did it affect you? what was your reaction (e.g. change gear, fishing spots?)

<Possible causes include: over fishing, pollution, weather or climate events, destructive fishing arts such as dinamite, industrial fishing, increase on number of fishers, infrastructure developments such as roads, pipelines; and invasive species>

<Questions 36-40 are to be asked only in case that abrupt events have been reported>

36. Since the occurrence of the event, have you changed your fishing practice? Yes __ No __

    36.1 *<If yes>* How did it change? ________________

    36.2 Since the event, do you spend more, less or the same time fishing? More __ Less __ The same __

    36.3 Since the event, did you change your fishing area? Yes __ No __

Future

37. Do you expect more sudden (more dramatic) changes in fish abundance in the future, or other aspects of the ecosystem such as mangroves and birds? Yes __ No __

38. In your opinion, what will be the main cause of future sudden (more dramatic) changes in fish abundance? ________________________________

39. Please imagine that the amount of fish decreases and you can only fish half of what you fish today. What would you do? *<Do not read the options first, just listen to the interviewee. If the person do not propose any options, read the options then:>*

39.1 *<Mark if you had to read the options>* __

39.2 Would you continue fishing? Yes __ No __

39.2.1 Why? ________________________________

39.3 Would you increase fishing effort to increase catch? Yes __ No __

39.3.1 How? ______________________________

39.4 Would you fish less? Yes __ No __

39.4.1 What would you do besides fishing? __________________________

39.5 Would you change the fishing area where you go fishing? Yes __ No __

39.5.1 Where would you fish instead? _____________________________

39.6 Would you change your fishing art?

39.6.1 Which one would you use? __________________________________

39.7 Would you stop fishing? Yes __ No __

39.7.1 What would you do instead of fishing? __________________________

39.8 Other? Which one: ______________________________

Cooperation and communication

Now let's talk about your connection or relation with other fishers…

40. Do you lend each other gear? Yes __ No __

41. If you have a problem with, for example, your boat engine or fish traders, who do you ask for help? _________________________________

Fishing cooperatives and formal organizations

42. Are you part of a fishing cooperative or similar organization? Yes __ No __

42.1 Which one? ____________________________

42.2 How often do you meet?
Once a year __ Twice a year __ Every month __ Every week __

42.3 When was the last time you meet? _____ (year)

42.4 What is your role in this organization?
Legal representative __ President __ Secretary __ Treasurer __ Active member __

42.4.1 Other? ____________________________

42.5 How do you benefit from being part of this organization? *<Do not read the options first, just listen to the interviewee. If the person do not propose any options, read the options then:>*

42.5.0 *<Mark if you had to read the options>* __

42.5.1 Better prices __

42.5.2 Conservation of fishing resources __

42.5.3 Support in times of low income __

42.5.4 Being respected and recognized as a member of the organization __

42.5.5 Pushed / forced of being a member by the government, local leaders, friends or family __

42.5.6 Social aspects (making friends, working with other fishers, fear of being excluded) __

42.5.7 More fishing and income __

42.5.8 Better quality of the fishing products

42.5.9 Other, which? ______________________________

Demographic and household information

43. Gender: Male __ Female __

44. Marital status:
Single __ Married __ co-habitation __ Divorced / widow __

45. Age: ___ years old

46. Education: How many years of formal education have you completed?
No formal education __ Elementary school __ Secondary school __ Higher education __ (Please specify) ___

47. Where you born here, in *<name of community>*? Yes __ No __

48. *<If not>* Where were you born? ________________

49. *<If not>* When did you move here? ______ (year)

50. *<If yes>* Have you always lived here? Yes __ No __

51. *<If no>* How long have you lived here? ______ (years / months) *<If the person mentioned being forcely displaced, ask how long did he/she lived elsewhere>* ____________

52. Now we will talk about ho do you feel today in *<name of place>*. I will reed a few options and ask you how in agreement or disagreemnt you are with the following sentences:

52.1 I miss *<name of place>* when I'm away
Strongly agree __ Agree __ Disagree __ Strongly disagree __

52.2 I don't feel that I belong to *<name of place>*.
Strongly agree __ Agree __ Disagree __ Strongly disagree __

52.3 I feel safe when I am in *<name of place>*.
Strongly agree __ Agree __ Disagree __ Strongly disagree __

52.4 I am proud when I am in *<name of place>*.
Strongly agree __ Agree __ Disagree __ Strongly disagree __

52.5 *<name of place>* is part of myself.
Strongly agree __ Agree __ Disagree __ Strongly disagree __

52.6 I would like to leave *<name of place>*.
Strongly agree __ Agree __ Disagree __ Strongly disagree __

52.7 I would like to be more committed with *<name of place>*.
Strongly agree __ Agree __ Disagree __ Strongly disagree __

52.8 My roots are in *<name of place>*.
Strongly agree __ Agree __ Disagree __ Strongly disagree __

52.9 I would like that my family and friends stay in *<name of place>* in the future.
Strongly agree __ Agree __ Disagree __ Strongly disagree __

Household composition:

53. Please list below the people who live with you in your house *<live under the same roof and eat from the same kitchen>* and their relationship with you:

< Please mark the ranking according to their contribution to the household income. Listen carefully and take note if people also work in fish processing or commercialization>*

53.1 Relationship?	53.2 Age	53.3 Job	53.4 Earnings rank*
Yourself			[1, 2, 3, 4]
			[1, 2, 3, 4]
			[1, 2, 3, 4]
			[1, 2, 3, 4]

54. If any of your relatives is gravely sick and needs an expensive treatment, how would you rise the necessary money? *<Example answers: selling something, bank loan, loan with friend, family or neighbour>*
____

55. *<if savings are not mentioned>* Do you have the possibility of requesting a loan? Yes __ No __

56. Do you have any additional comment (e.g. about fishing, the experiment, or any recommendation for your grandchildren) ______________________________

Table S1: Clustered and robust standard errors estimation for individual extraction with White method and (1) HC1, (2) HC2, (3) HC3, (4) HC4 weighting schemes, and (5) Newey and West method with HC4 scheme.

	(1)	(2)	(3)	(4)	(5)
Constant	-0.12	-0.12	-0.12	-0.12	-0.12
	(0.19)	(0.19)	(0.19)	(0.19)	(0.18)
Treatment: Threshold	-0.26	-0.26	-0.26	-0.26	-0.26
	(0.16)	(0.16)	(0.16)	(0.16)	(0.18)
Treatment: Risk	0.09	0.09	0.09	0.09	0.09
	(0.20)	(0.20)	(0.20)	(0.20)	(0.24)
Treatment: Uncertainty	-0.35^{**}	-0.35^{**}	-0.35^{**}	-0.35^{**}	-0.35^{**}
	(0.16)	(0.16)	(0.16)	(0.16)	(0.17)
Part	-0.03	-0.03	-0.03	-0.03	-0.03
	(0.11)	(0.11)	(0.11)	(0.11)	(0.11)
Round	-0.01	-0.01	-0.01	-0.01	-0.01
	(0.01)	(0.01)	(0.01)	(0.01)	(0.01)
Stock size	0.08^{***}	0.08^{***}	0.08^{***}	0.08^{***}	0.08^{***}
	(0.003)	(0.003)	(0.003)	(0.003)	(0.003)
Threshold * part	-0.20^{*}	-0.20^{*}	-0.20^{*}	-0.20^{*}	-0.20
	(0.11)	(0.11)	(0.11)	(0.11)	(0.12)
Risk * part	-0.50^{***}	-0.50^{***}	-0.50^{***}	-0.50^{***}	-0.50^{***}
	(0.14)	(0.14)	(0.14)	(0.14)	(0.16)
Uncertainty * part	-0.22^{**}	-0.22^{**}	-0.22^{**}	-0.22^{**}	-0.22^{*}
	(0.11)	(0.11)	(0.11)	(0.11)	(0.12)

Note: $^{*}p<0.1$; $^{**}p<0.05$; $^{***}p<0.01$

Table S2: Clustered and robust standard errors estimation for proportion of available stock with White method and (1) HC1, (2) HC2, (3) HC3, (4) HC4 weighting schemes, and (5) Newey and West method with HC4 scheme.

	(1)	(2)	(3)	(4)	(5)
Constant	0.09^{***}	0.09^{***}	0.09^{***}	0.09^{***}	0.09^{***}
	(0.01)	(0.01)	(0.01)	(0.01)	(0.01)
Treatment: Threshold	-0.02	-0.02	-0.02	-0.02	-0.02
	(0.02)	(0.02)	(0.02)	(0.02)	(0.01)
Treatment: Risk	-0.004	-0.004	-0.004	-0.004	-0.004
	(0.02)	(0.02)	(0.02)	(0.02)	(0.02)
Treatment: Uncertainty	-0.02	-0.02	-0.02	-0.02	-0.02^{*}
	(0.01)	(0.01)	(0.02)	(0.01)	(0.01)
Part	-0.004	-0.004	-0.004	-0.004	-0.004
	(0.01)	(0.01)	(0.01)	(0.01)	(0.01)
Round	0.001^{***}	0.001^{***}	0.001^{***}	0.001^{***}	0.001^{***}
	(0.0004)	(0.0004)	(0.0004)	(0.0004)	(0.0004)
Threshold * part	-0.01^{*}	-0.01^{*}	-0.01^{*}	-0.01^{*}	-0.01^{**}
	(0.01)	(0.01)	(0.01)	(0.01)	(0.01)
Risk * part	-0.03^{***}	-0.03^{***}	-0.03^{***}	-0.03^{***}	-0.03^{***}
	(0.01)	(0.01)	(0.01)	(0.01)	(0.01)
Uncertainty * part	-0.02^{**}	-0.02^{**}	-0.02^{**}	-0.02^{**}	-0.02^{**}
	(0.01)	(0.01)	(0.01)	(0.01)	(0.01)

Note: $^{*}p<0.1$; $^{**}p<0.05$; $^{***}p<0.01$

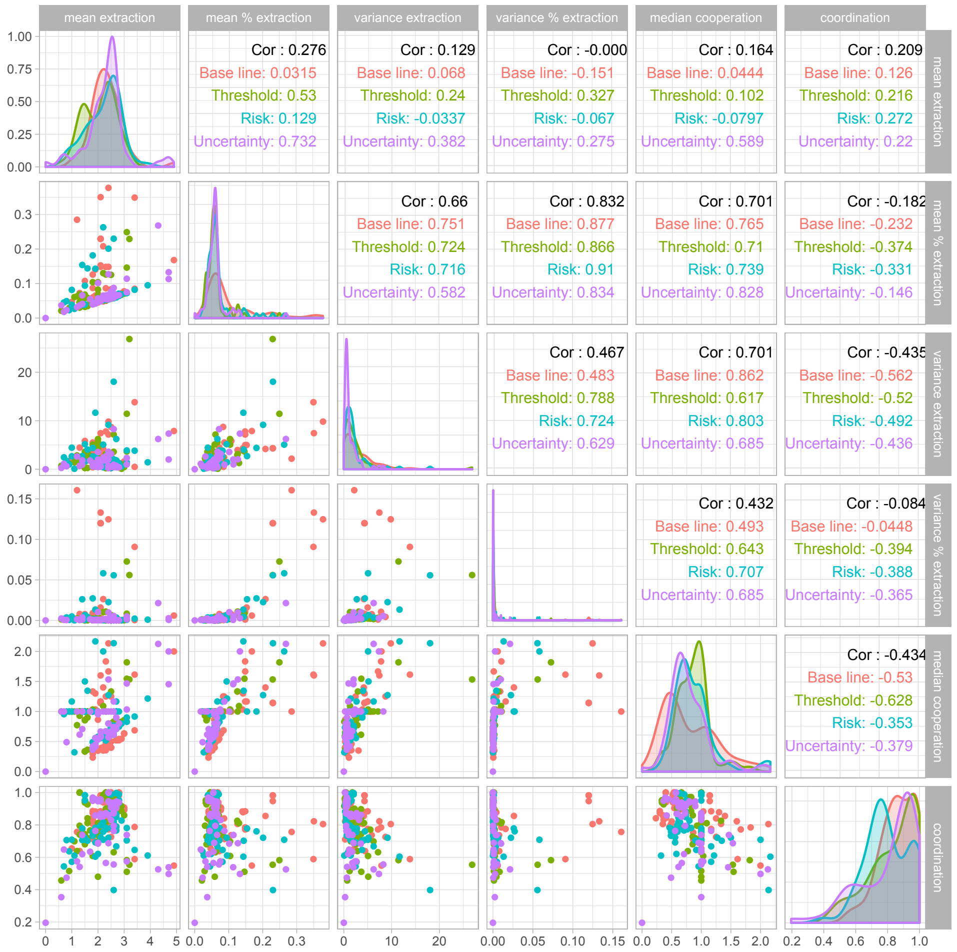

Figure S1: **Correlations between response variables.** Correlation coefficients are calculated by variable and by treatment.

Table S3: Clustered and robust standard errors estimation for cooperation with White method and (1) HC1, (2) HC2, (3) HC3, (4) HC4 weighting schemes, and (5) Newey and West method with HC4 scheme.

	(1)	(2)	(3)	(4)	(5)
Constant	0.79^{***}	0.79^{***}	0.79^{***}	0.79^{***}	0.79^{***}
	(0.13)	(0.13)	(0.13)	(0.13)	(0.16)
Treatment: Threshold	−0.21	−0.21	−0.21	−0.21	−0.21
	(0.18)	(0.18)	(0.18)	(0.18)	(0.20)
Treatment: Risk	−0.08	−0.08	−0.08	−0.08	−0.08
	(0.18)	(0.18)	(0.18)	(0.18)	(0.20)
Treatment: Uncertainty	−0.24	−0.24	−0.24	−0.24	−0.24
	(0.16)	(0.16)	(0.16)	(0.16)	(0.18)
Part	-0.34^{***}	-0.34^{***}	-0.34^{***}	-0.34^{***}	-0.34^{***}
	(0.06)	(0.06)	(0.06)	(0.06)	(0.08)
Round	0.05^{***}	0.05^{***}	0.05^{***}	0.05^{***}	0.05^{***}
	(0.004)	(0.004)	(0.004)	(0.004)	(0.004)
Threshold * part	0.19^{***}	0.19^{***}	0.19^{***}	0.19^{***}	0.19^{**}
	(0.07)	(0.07)	(0.07)	(0.07)	(0.08)
Risk * part	0.06	0.06	0.06	0.06	0.06
	(0.07)	(0.07)	(0.07)	(0.07)	(0.08)
Uncertainty * part	0.13^{*}	0.13^{*}	0.13^{*}	0.13^{*}	0.13^{*}
	(0.07)	(0.07)	(0.07)	(0.07)	(0.07)
Note:					*p$<$0.1; **p$<$0.05; ***p$<$0.01

Table S4: Original regression models as shown in Fig 3. Dependend variables are (1) mean extraction, (2) mean proportion of extraction, (3) median cooperation, (4) variance of cooperation, (5) variance of extraction, (6) variance of the proportion of extraction, and (7) coordination.

	Dependent variable:						
	(1)	(2)	(3)	(4)	(5)	(6)	(7)
Constant	1.38^{***}	0.08^{***}	0.87^{***}	0.98^{***}	4.01^{***}	0.02^{*}	0.23^{**}
	(0.26)	(0.02)	(0.16)	(0.33)	(0.76)	(0.01)	(0.10)
Treatment: Threshold	−0.19	-0.02^{*}	0.09	0.18	−0.06	−0.01	−0.02
	(0.12)	(0.01)	(0.09)	(0.28)	(0.71)	(0.01)	(0.02)
Treatment: Risk	−0.15	-0.03^{**}	0.06	0.09	−0.69	−0.01	−0.02
	(0.11)	(0.01)	(0.10)	(0.34)	(0.51)	(0.01)	(0.02)
Treatment: Uncertainty	−0.03	-0.03^{**}	0.05	0.19	-0.97^{*}	−0.01	-0.08^{***}
	(0.12)	(0.01)	(0.08)	(0.26)	(0.53)	(0.01)	(0.03)
Place: B	-0.22^{*}	0.003	0.03	−0.07	1.78^{**}	0.001	-0.07^{***}
	(0.13)	(0.01)	(0.10)	(0.46)	(0.66)	(0.01)	(0.02)
Place: C	0.25	−0.01	−0.11	−0.11	0.72	−0.003	−0.01
	(0.17)	(0.01)	(0.09)	(0.26)	(0.51)	(0.01)	(0.03)
Place: D	0.29^{**}	−0.02	-0.19^{**}	−0.52	−0.26	−0.01	−0.03
	(0.13)	(0.01)	(0.09)	(0.36)	(0.50)	(0.01)	(0.02)
Education	0.001	−0.001	−0.003	−0.03	-0.12^{**}	−0.0002	−0.001
	(0.01)	(0.001)	(0.01)	(0.03)	(0.05)	(0.0003)	(0.001)
Frequency of bad fishing days	0.04^{*}	0.001	0.005	−0.04	0.11	−0.0003	−0.002
	(0.02)	(0.002)	(0.01)	(0.05)	(0.09)	(0.001)	(0.003)
Expectation of fishing children	−0.07	0.005	−0.05	−0.11	−0.48	0.01	0.01
	(0.08)	(0.01)	(0.05)	(0.15)	(0.40)	(0.01)	(0.01)
Fishing art sharing	-0.20^{*}	−0.01	−0.05	−0.12	0.29	−0.003	0.01
	(0.11)	(0.01)	(0.05)	(0.16)	(0.46)	(0.003)	(0.01)
Group fishing	0.02	0.002	0.03	−0.08	0.03	−0.002	0.01
	(0.11)	(0.01)	(0.06)	(0.25)	(0.50)	(0.004)	(0.01)
Risk aversion	0.01	0.0001	−0.003	0.06	−0.11	0.0004	0.004
	(0.02)	(0.002)	(0.01)	(0.05)	(0.08)	(0.001)	(0.003)
Ambiguity aversion	−0.01	0.002^{*}	0.01	0.01	0.03	0.001	0.004
	(0.02)	(0.001)	(0.01)	(0.03)	(0.07)	(0.001)	(0.003)
Rounds with agreements	0.20	-0.03^{**}	-0.33^{**}	-0.69^{*}	-2.13^{***}	−0.01	0.20^{***}
	(0.14)	(0.01)	(0.12)	(0.37)	(0.69)	(0.01)	(0.04)
Part 1 variable (1)	0.29^{***}						
	(0.08)						
Part 1 variable (2)		0.53^{***}					
		(0.14)					
Part 1 variable (3)			0.27^{***}				
			(0.06)				
Part 1 variable (4)				0.24			
				(0.20)			
Part 1 variable (5)					0.06		
					(0.04)		
Part 1 variable (6)						0.22	
						(0.17)	
Part 1 variable (7)							0.62^{***}
							(0.11)
Observations	236	236	236	236	236	236	236
R^2	0.31	0.54	0.43	0.31	0.41	0.30	0.78
Adjusted R^2	0.26	0.51	0.39	0.26	0.37	0.25	0.77
Residual Std. Error	0.58	0.04	0.29	1.31	2.34	0.02	0.08
F Statistic	6.60^{***}	17.21^{***}	10.94^{***}	6.58^{***}	10.34^{***}	6.18^{***}	53.24^{***}

Note: *p<0.1; **p<0.05; ***p<0.01
Clustered robust standard errors and confidence intervals were calculated with the CR2 estimator

Table S5: Modified model without place terms. Dependend variables are (1) mean extraction, (2) mean proportion of extraction, (3) median cooperation, (4) variance of cooperation, (5) variance of extraction, (6) variance of the proportion of extraction, and (7) coordination.

	Dependent variable:						
	(1)	(2)	(3)	(4)	(5)	(6)	(7)
Constant	1.16^{***}	0.07^{***}	0.88^{***}	0.90^{**}	4.68^{***}	0.02^{***}	0.16^{***}
	(0.22)	(0.01)	(0.10)	(0.39)	(0.71)	(0.01)	(0.04)
Treatment: Threshold	-0.22^{*}	-0.02^{***}	0.10^{*}	0.20	0.05	-0.01^{***}	−0.02
	(0.12)	(0.01)	(0.06)	(0.25)	(0.47)	(0.004)	(0.02)
Treatment: Risk	−0.15	-0.03^{***}	0.06	0.10	−0.70	-0.01^{***}	−0.02
	(0.11)	(0.01)	(0.06)	(0.24)	(0.45)	(0.004)	(0.02)
Treatment: Uncertainty	−0.04	-0.03^{***}	0.05	0.19	-0.92^{**}	-0.01^{***}	-0.08^{***}
	(0.11)	(0.01)	(0.06)	(0.25)	(0.46)	(0.004)	(0.02)
Education	0.01	−0.001	−0.01	−0.03	-0.10^{**}	−0.0003	−0.001
	(0.01)	(0.001)	(0.01)	(0.02)	(0.04)	(0.0004)	(0.001)
Frequency of bad fishing days	0.04^{*}	0.001	0.004	−0.03	0.10	−0.0003	−0.0002
	(0.02)	(0.002)	(0.01)	(0.05)	(0.09)	(0.001)	(0.003)
Expectation of fishing children	−0.11	0.01	−0.02	−0.05	−0.44	0.01^{*}	0.01
	(0.10)	(0.01)	(0.05)	(0.22)	(0.41)	(0.003)	(0.01)
Fishing art sharing	−0.08	-0.01^{**}	-0.09^{*}	−0.17	−0.14	−0.005	0.03^{**}
	(0.09)	(0.01)	(0.05)	(0.20)	(0.38)	(0.003)	(0.01)
Group fishing	0.06	0.0004	0.01	−0.14	0.14	−0.003	0.001
	(0.11)	(0.01)	(0.05)	(0.23)	(0.43)	(0.004)	(0.01)
Risk aversion	0.004	0.0002	−0.003	0.06	−0.05	0.0005	0.002
	(0.03)	(0.002)	(0.01)	(0.05)	(0.10)	(0.001)	(0.003)
Ambiguity aversion	−0.02	0.002	0.01	0.002	0.06	0.001	0.002
	(0.03)	(0.002)	(0.01)	(0.06)	(0.10)	(0.001)	(0.003)
Rounds with agreements	0.35^{***}	-0.03^{***}	-0.36^{***}	-0.70^{**}	-2.68^{***}	-0.01^{*}	0.21^{***}
	(0.13)	(0.01)	(0.07)	(0.28)	(0.53)	(0.004)	(0.02)
Part 1 variable (1)	0.31^{***}						
	(0.04)						
Part 1 variable (2)		0.54^{***}					
		(0.05)					
Part 1 variable (3)			0.28^{***}				
			(0.04)				
Part 1 variable (4)				0.23^{***}			
				(0.03)			
Part 1 variable (5)					0.05^{***}		
					(0.01)		
Part 1 variable (6)						0.22^{***}	
						(0.04)	
Part 1 variable (7)							0.65^{***}
							(0.05)
Observations	236	236	236	236	236	236	236
R^2	0.24	0.53	0.38	0.29	0.35	0.28	0.76
Adjusted R^2	0.20	0.50	0.35	0.26	0.32	0.24	0.75
Residual Std. Error	0.60	0.04	0.30	1.32	2.45	0.02	0.08
F Statistic	5.99^{***}	20.64^{***}	11.55^{***}	7.71^{***}	10.01^{***}	7.31^{***}	58.82^{***}

Note: *p<0.1; **p<0.05; ***p<0.01
Clustered robust standard errors and confidence intervals were calculated with the CR2 estimator.

Table S6: Modified model with only treatment and place. Dependend variables are (1) mean extraction, (2) mean proportion of extraction, (3) median cooperation, (4) variance of cooperation, (5) variance of extraction, (6) variance of the proportion of extraction, and (7) coordination.

	Dependent variable:						
	(1)	(2)	(3)	(4)	(5)	(6)	(7)
Constant	1.44^{***}	0.06^{***}	0.63^{***}	0.39^{*}	2.29^{***}	0.02^{***}	0.19^{***}
	(0.15)	(0.01)	(0.06)	(0.21)	(0.40)	(0.003)	(0.04)
Treatment: Threshold	-0.23^{**}	-0.03^{***}	0.09^{*}	0.06	-0.22	-0.01^{***}	0.001
	(0.10)	(0.01)	(0.05)	(0.22)	(0.42)	(0.004)	(0.02)
Treatment: Risk	-0.12	-0.03^{***}	0.07	0.10	-0.70	-0.01^{***}	-0.005
	(0.10)	(0.01)	(0.05)	(0.22)	(0.42)	(0.004)	(0.02)
Treatment: Uncertainty	0.03	-0.03^{***}	0.01	0.03	-1.28^{***}	-0.01^{***}	-0.05^{***}
	(0.10)	(0.01)	(0.05)	(0.23)	(0.42)	(0.004)	(0.02)
Place: B	-0.20^{**}	0.01	0.09	0.13	1.80^{***}	0.003	-0.08^{***}
	(0.10)	(0.01)	(0.05)	(0.22)	(0.42)	(0.004)	(0.02)
Place: C	0.35^{***}	-0.02^{**}	-0.18^{***}	-0.42^{*}	-0.45	-0.01^{**}	0.03^{*}
	(0.10)	(0.01)	(0.05)	(0.22)	(0.42)	(0.004)	(0.02)
Place: D	0.22^{**}	-0.02^{***}	-0.16^{***}	-0.54^{**}	-0.60	-0.01^{**}	-0.02
	(0.10)	(0.01)	(0.05)	(0.22)	(0.42)	(0.004)	(0.02)
Part 1 variable (1)	0.28^{***}						
	(0.04)						
Part 1 variable (2)		0.57^{***}					
		(0.05)					
Part 1 variable (3)			0.33^{***}				
			(0.04)				
Part 1 variable (4)				0.25^{***}			
				(0.03)			
Part 1 variable (5)					0.06^{***}		
					(0.01)		
Part 1 variable (6)						0.23^{***}	
						(0.04)	
Part 1 variable (7)							0.83^{***}
							(0.04)
Observations	256	256	256	256	256	256	256
R^2	0.26	0.51	0.34	0.27	0.32	0.27	0.69
Adjusted R^2	0.24	0.49	0.33	0.25	0.31	0.25	0.68
Residual Std. Error	0.58	0.04	0.30	1.27	2.39	0.02	0.09
F Statistic	12.43^{***}	36.51^{***}	18.62^{***}	13.36^{***}	16.99^{***}	13.10^{***}	78.03^{***}

Note: $^{*}p<0.1$; $^{**}p<0.05$; $^{***}p<0.01$
Clustered robust standard errors and confidence intervals were calculated with the CR2 estimator.
