# Supplementary material for: Cooperation in the face of thresholds, risk, and uncertainty: Experimental evidence in fisher communities from Colombia
Source: PLoS One. 2020 Dec 28;15(12):e0242363. doi: 10.1371/journal.pone.0242363 (PMC7769437; doi:10.1371/journal.pone.0242363)
Supplement: S2 File — (PDF) [file pone.0242363.s002.pdf]

Instrucciones

###
Texto normal: se lee en voz alta a los participantes
< *texto en cursiva: acciones del experimentalista o asistentes* >
" - - - " significa hacer una pausa cuando lea las instrucciones
Lo que este subrayado es texto donde necesita hacer énfasis.

Bienvenidos y gracias por participar de esta actividad! Usted ha sido invitado a participar en este ejercicio, que forma parte de un estudio sobre el manejo de los recursos naturales. En este estudio, es muy importante la participación de personas como usted, que por su trabajo diario están en contacto con el manejo de los recursos pesqueros. Nosotros somos investigadores del Instituto Beijer de Economía Ecológica y la Universidad del Magdalena. Mi nombre es Juan Carlos Rocha y todos los miembros de nuestro equipo tienen una escarapela con cada nombre escrito. Gracias a su participación en el juego podremos aprender todos de este estudio. Durante la actividad vamos a jugar un juego de decisiones y después les haremos una encuesta a cada uno de ustedes. En total la actividad tomara aproximadamente 3 horas de su tiempo.

Como se anunció anteriormente, usted recibirá un mínimo de 15.000 pesos por participar de esta actividad. Esto significa que sin importar el resultado del juego, usted seguro recibirá 15.000 pesos. Dependiendo de las decisiones que usted tome en el juego usted podrá ganar un dinero extra. Usted recibirá su dinero al final de la actividad - después de hacerle la encuesta- y su pago será en privado.

Usted se preguntará: Por que usamos dinero? — Nosotros NO vemos el dinero que usted gana como un pago por su participación en la actividad, ni tampoco la razón por la que usted esta aquí. — Nosotros usamos dinero porque el ejercicio requiere que usted tome decisiones con consecuencias para su bolsillo, como en la vida real. Por favor, tenga en cuenta que si usted ha participado en otro juego de toma de decisiones en el pasado, el juego que jugaremos hoy es completamente diferente.

Antes de empezar, nos gustaría que firmara una autorización de pago que confirma que usted quiere participar de manera voluntaria. A continuación les leeré la autorización para aquellas personas que olvidaron sus gafas o tengan dificultad para leer. *<leer consentimiento>* . En resumen la autorización dice que usted esta aquí de manera voluntaria. La autorización también es un acuerdo entre usted y nosotros de que usted tomara parte de toda la actividad. Si usted o uno de los miembros de su grupo decide irse antes de terminar la actividad, ni usted o los miembros de su grupo podrán terminar la actividad y por tanto no recibirán pago alguno. Por favor firmemos la autorización ahora y los asistentes pasarán recogiéndola.

Explicar acceso común a un recurso pesquero

En este juego queremos que ustedes se imaginen que tienen acceso a un recurso pesquero comunitario.

< Señale el recipiente en la mesa en frente de ellos con las fichas que representan pescado>

Aunque en la realidad es imposible saber exactamente cuanto pescado hay en el mar o en la ciénaga, — en este juego vamos a suponer que nosotros sabemos exactamente cuanto pescado hay. También asumimos que solo hay una especie de pescado en la zona de pesca. Cada uno de ustedes puede pescar los pescados de esta pesquería común.

Explicar la toma de decisiones

El juego tiene dos etapas, cada etapa durará varias rondas y en cada una de ellas usted tomara una decisión individual sobre cuanto quiere usted pescar. Nosotros estaremos atentos de que sus compañeros no puedan ver sus decisiones. Esto significa que usted puede hablar de lo que quiera incluso de cuánto pescado quiere sacar.

< Introduzca y muestre las tarjetas de decision. Expliques que escribir y en donde.. Muestre la tarjeta de decision y explique: >

Cada ronda del juego equivale a una faena de pesca. En la columna izquierda “Pesca Individual” *< señálela>* usted indica cuanto pescado quiere capturar en la faena. Nuestros asistentes están aquí para ayudarle si usted lo necesita, en caso que tenga dificultades para leer, escribir, o alguna pregunta respecto al juego. En cada faena usted puede escoger la cantidad de pescado disponible en la ronda o no pescar lo que equivale a cero. Tenga en cuenta que sus decisiones afectan su bienestar individual así como el bienestar de la comunidad.

Por cada pescado que usted capture, usted gana 500 pesos. Así, — por ejemplo— si usted pesca 10 pescados, usted ganaría 10 * 500 = 5.000 pesos. (Adicionales a los 15.000 pesos que ya se gano por participar de la actividad). Usted puede llevar las cuentas de sus ganancias individuales en cada faena en la segunda columna de su tarjeta de decisión coloreada en gris *< señálela>* . Nosotros le daremos calculadoras y si necesita ayuda no dude en llamar a alguno de los asistentes para que le ayude con las cuentas.

Una vez ustedes hayan tomado su decisión nosotros recogeremos sus tarjetas de decisión al final de cada ronda. —por favor cierre su tarjeta de decisión cuando se la entregue al asistente. Nosotros le ayudaremos a mantener su privacidad y la de sus compañeros.

Una vez hayamos colectado sus tarjetas de decisión, nosotros calcularemos cuantos pescados estarán disponibles en la siguiente ronda y le informaremos diciendo en voz alta y escribiéndolo aquí *< señale la tabla>* en la tabla.

El recurso pesquero será afectado por la extracción que usted y los otros miembros de su grupo hagan, así como la capacidad del recurso de reproducirse.

Explicar como se reproduce el pescado dependiendo del tamaño de la población

Ahora les voy a explicar como se reproduce el pescado, usando ésta ilustración *< señalar la ilustración con los peces >*. La población de pescado crece (o no) en cada faena de pesca. El crecimiento depende de cuanto pescado hay — es decir el tamaño de la población o el cardumen. Comenzaremos con 50 pescados en la primera ronda. Usted vera que …

< explicar la ilustración >

— Si hay entre 46 - 50 pescados, el cardumen no crece. El numero de pescados disponibles en este caso es el mismo numero de pescados para la siguiente ronda. *< Si preguntan porque la respuesta es: Hay alta competencia por comida y hay mucho pescado en relación con el tamaño del sitio de pesca>*

— Si hay entre 35 - 45 pescados, en la siguiente ronda se reproducirán 5 mas. *< si los participantes preguntan por qué, la respuesta es: hay competencia por comida y espacio, hay bastante pescado en relación con el tamaño del sitio de pesca. Así que el recurso no crece tanto como cuando esta entre 24 -34 pescados>*

— Si hay entre 24-34 pescados, habrá 10 pescados mas en la siguiente ronda. *< si los participantes le preguntan por qué, la respuesta es: Hay pescados suficientes de tal manera que pueden encontrar pareja, pero no demasiados como para que compitan por comida y espacio>*

— Si hay entre 5 -19 pescados, habrá 5 pescados mas en la siguiente ronda. *< si los participantes le preguntan por qué, la respuesta es: hay muy pocos pescados y les queda difícil encontrar pareja>*

— Si hay menos de 5 pescados, el recurso no crecerá (al igual que con tamaño de recurso entre 46-50 pescados) *< si los participantes le preguntan por qué, la respuesta es: hay muy pocos peces y no pueden encontrar pareja>*

Como mencioné anteriormente, este juego dura varias rondas y tiene dos partes nosotros le anunciaremos cuando termine la primera parte y le explicaremos de que se trata la segunda parte. — Nosotros no le diremos el numero exacto de rondas que usted jugará en cada parte del juego. Sin embargo, usted sabe que jugaremos por un máximo de dos horas. — Recuerde: si no hay mas pescado disponible, esa parte del juego se acaba y usted no ganará más dinero por el momento. Siempre y cuando haya peces para pescar, la parte del juego continuará y usted podrá ganar dinero. También recuerde: sus decisiones afectan sus ganancias individuales pero también afectan el bienestar colectivo del grupo con el que usted comparte la pesquería.

Tienen alguna pregunta antes de continuar con los ejemplos?

< si alguien pregunta sobre como compartir un nivel de extracción mayor al pescado disponible en el recurso, repartiremos lo disponible proporcionalmente a lo que cada uno pescó>

Ejemplos

< Use el recipiente y las fichas. Haga a los pescadores participen, preguntes cuanto creen que se reproducen los peces de ronda a ronda con los ejemplos>

Tendremos 50 peces al comienzo del juego. Ahora, supongamos que entre todos los miembros del grupo se capturan 12 peces (por ejemplo usted pescó 4, usted 3, usted también 3, y usted 2). *< saque 12 fichas del recipiente>*. Entonces nos quedan 38 peces que son 50 menos 12. Ahora podemos comprobar aquí en la tabla *< señálela>* la reproducción de los peces cuando el recurso tiene 38. Cuanto cree que se reproducen los peces?? *< pregunte a los participantes>* -- Si, exacto! se reproducen 5 mas. *< ponga 5 fichas en el recipiente>*. Así en la ronda siguiente habrán 43 peces (50 -12 + 5) y este es el numero que les comunicaremos en voz alta y escribiremos aquí *< señalar la tabla del tamaño del recurso para cada ronda>*

Hagamos otro ejemplo…
Ahora tenemos 43 peces en el recipiente. [Pedir que un pescador sea el asistente] Si ustedes pescan 13 en total *< saque 13 fichas>* entonces nos quedan 30 peces y la población crecerá con cuantos peces más en la siguiente ronda? *<pregúntele a los pescadores>*. Exacto! 10 peces más. *< ponga 10 fichas en el recipiente>* así que para la siguiente ronda habrán disponibles 40 peces.

Si la extracción grupal excede en numero de peces disponibles en una ronda, la parte del juego en la que estemos termina dado que no hay mas peces disponibles para pescar. En dado caso, el pescado restante se divide de manera proporcional.

< Ejemplo en caso de que pregunten: Por ejemplo, si hay solo 10 peces disponibles pero la extracción colectiva es de 100 pescados (supongamos 30, 20, 30, 20), los pescados que quedaron serán divididos proporcionalmente a lo que usted quizo pescar. Así si su intención de pesca eran 30 peces de los 100 que como grupo intentaron extraer (30%), entonces usted recibirá el 30% de los peces que estaban disponibles en realidad, esto es 3 peces (30% de 10)>

— Ahora usted sabe como indicar su decisión individual en su tarjeta de decisión en cada ronda o faena— y usted también sabe como calcular el tamaño de la población para la siguiente ronda: usted sencillamente resta la captura colectiva del tamaño de la población del cardumen, y luego le suma la reproducción de pescado respectivamente. Recuerde, nosotros llevaremos las cuentas del tamaño del cardumen por usted en cada ronda, pero nosotros queremos que usted entienda claramente como funciona. Alguna pregunta?

Ahora vayamos a la tercer columna de la tarjeta de decisión. En la primer y segunda columna corresponden a la pesca individual y sus ganancias <señalar>. Nos gustaría que escribiera cuántos pescados cree usted que habrá en la siguiente ronda — lo que usted piensa que escribiremos en la tabla para comenzar la siguiente ronda. Ustedes deciden cuanto pescar en la faena, hacen sus cuentas de las ganancias individuales o le piden a un asistente que les ayude con las cuentas, y también hacen su especulación sobre lo que pasara en la siguiente ronda con el cardumen. Luego nosotros hacemos las cuentas y haremos publico lo que pasa con el recurso colectivo pero no revelamos que pescó cada quien, solamente lo que paso a nivel de grupo. Así usted

podrá comparar lo que usted esperaba que sucediera (la tercer columna) con lo que pasa en la realidad (la tabla publica del estado del cardumen).

Comunicación

…durante las dos partes del juego y desde el comienzo ustedes pueden hablar entre ustedes. Esto significa que usted puede hablar de lo que quiera sobre sus decisiones, sobre las reglas, estrategias e incluso de cuánto pescado quiere sacar. Ustedes no pueden hacer ninguna amenaza, arreglo o pagos durante o después de la actividad. Ustedes tienen dos minutos entre rondas para discutir con los otros miembros de su grupo cuanto quieren pescar, después de esos dos minutos podrá escribir su decisión individual. Nosotros llevaremos cuenta del tiempo y les informaremos cuando necesiten entregar sus tarjetas de decisión.

Tienen alguna pregunta?
-- Por favor recuerde, — en caso de alguna pregunta durante el juego, no dude en pregúntenos y nosotros le aclararemos sus dudas.

División en grupos

Ahora ustedes formaran grupos de 4 personas.

< Dividir los grupos. Pedir a los asistentes que repartan escarapelas al azar. Cada sujeto toma al azar una papeleta de color que le indica en que grupo estará situado con en número de jugador (1-4) que tendrá en el grupo. Así, por ejemplo, si tenemos tres grupos jugando al mismo tiempo podríamos tener algo así como Azul {1,2,3,4}, Verde {1,2,3,4}, y Amarillo {1,2,3,4}. Tenga en cuenta que las personas que comparten hogar o amigos cercanos deben quedar en grupos diferentes en la medida de lo posible>

Ya que los grupos están formados, podría por favor levantar su mano si alguno de los miembros de su grupo vive con usted?

<De ahora en adelante cada grupo esta con un monitor propio. El monitor resume las reglas del juego antes de comenzar los ejercicios de práctica >

Buenos días / tardes. Mi nombre es <su nombre> y nuestro asistente se llama <nombre> y nosotros les ayudaremos a jugar el juego. Ahora ustedes son un grupo de 4 pescadores que comparten el mismo sitio de pesca con 50 pescados. En frente de ustedes tiene un folder con sus tarjetas de decisión, una calculadora y un esfero. Si necesitan ayuda para escribir o hacer sus cuentas nuestro asistente y yo estamos aquí para ayudarles.

Rondas de práctica

Antes de comenzar la primera parte del juego haremos unos ejercicios de practica para asegurarnos que todos hayamos entendido bien las instrucciones. Por favor recuerde: usted no gana dinero durante las rondas de practica. Durante el ejercicio de práctica nosotros no revelamos quien extrajo que, solo lo que “alguien extrajo” y nosotros haremos las cuentas del tamaño poblacional públicamente de tal manera que todos puedan seguirnos. Ahora tienen dos minutos que pueden usar para discutir sus decisiones, después de este tiempo deberán escribir su decisión y nosotros recogeremos las carpetas.

<Recogemos las tarjetas de decisión y se las llevamos a Caroline. Ella le entregara un papel con las capturas individuales en desorden para que usted las pueda leer públicamente. Use el recipiente y fichas sobre la mesa, extraiga y reponga fichas respectivamente>

Bueno, en el ejercicio alguien pescó … *<saque pescados del mar a medida que lee>,* otro pescador pescó…, otro … y el ultimo ….*<compruebe con los pescadores cuanto pescado queda en el mar>.* Por tanto, el cardumen quedó con XX peces. Entonces, cuántos peces se reproducirán para la siguiente ronda?

<Déjelos responder. Cuando hayan calculado el nuevo tamaño del cardumen escríbalo en la cartelera grande en frente de la ronda P1 (de practica). Repita la ronda de practica 2 o 3 veces y asegúrese que todos hayan entendido>

Alguna pregunta?

Si no, comencemos la primera parte del juego.
— Esto significa que desde ahora usted ganará dinero de acuerdo a sus decisiones. En caso de que usted escuche a los otros grupos jugar, usted debe saber que lo que pasa en otros grupos no tiene nada que ver con lo que pasa en su juego. Ellos puede que terminen antes, pero eso no significa que el juego en el que usted participa también terminará. Antes de comenzar la primera parte del juego de verdad, les resumo las reglas del juego:

- Ustedes cuatro comparten una zona pesquera comunitaria
- En cada ronda usted tomará una decisión individual sobre cuantos peces quiere pescar. Usted puede escoger cuanto pescado haya disponible o no pescar (cero). Por cada pescado capturado usted gana 500 pesos. Usted también adivinará cuantos pescados quedaran para la siguiente faena.
- Sus decisiones afectan su bienestar individual pero también afectan el recurso de toda la comunidad.
- Siempre y cuando haya peces disponibles, esta parte del juego continuará y ustedes podrán seguir ganando plata. Si el recurso colapsa esta parte del juego termina.
- Nosotros no le diremos cuantas rondas va a jugar en esta parte del juego.
- La reproducción del pescado depende de cuanto pescado queda en la zona de pesca *<señale la ilustración y recuérdeles las tablas>*
- Ustedes no pueden mostrarle lo que escriben en sus tarjetas de decisión a los otros pescadores
- Recuerde, ustedes pueden hablar durante todo el juego, Esto significa que usted puede hablar de lo que quiera incluso de cuánto pescado quiere sacar por un máximo de 2 minutos entre cada ronda, después de esos dos minutos usted debe escribir su decisión.

¿Tienen alguna pregunta?

inicia primera parte del juego

El lugar de pesca común empieza teniendo 50 peces, ahora tienen dos minutos que pueden usar para discutir sus decisiones, después de este tiempo deberán escribir su decisión y nosotros recogeremos las carpetas

< después de cada ronda, usted anuncia el nuevo tamaño poblacional>

El nuevo tamaño del recurso es […]

< el asistente del grupo ajusta el tamaño del cardumen respectivamente - Tenga en cuenta!! solo actualice el tamaño de la población, no revele la extracción del grupo cuando ponga o quite fichas>

< Ejemplo en caso de que pregunten: Por ejemplo, si hay solo 10 peces disponibles pero la extracción colectiva es de 100 pescados (supongamos 30, 20, 30, 20), los pescados que quedaron serán divididos proporcionalmente a lo que usted quizo pescar. Así si su intención de pesca eran 30 peces de los 100 que como grupo intentaron extraer (30%), entonces usted recibirá el 30% de los peces que estaban disponibles en realidad, esto es 3 peces (30% de 10)>

Introducción a los tratamientos

Incertidumbre

[después de la ronda 6]

Ahora juguemos la segunda parte. En esta ronda, queremos informarle que el tamaño de la población para la siguiente ronda es […]. <escriba el tamaño de la población de la última ronda de la primera parte del juego>También queremos informarle que un evento climático — con consecuencias negativas y duraderas para el recurso pesquero que ustedes manejan puede ocurrir.

Este tipo de evento climático es impredecible. Esto significa que usted no sabe si el evento va a ocurrir o cuando. Puede ocurrir en la siguiente ronda, en cuatro rondas, — o nunca…

En el caso de que el evento ocurra, — la reproducción del pescado se verá afectada, se reducirá por debajo del tamaño poblacional de 28 peces, como pueden ver aquí *<señale la nueva ilustración con dinámica de umbral>* la reproducción será únicamente de un pez para tamaños poblacionales entre 2 y 27 pescados. Para los demás tamaños poblacionales la reproducción será la misma, entre 28-34 serán 10 pescados, entre 35-45 serán 5 pescados, como pueden ver aquí <señalar>. Los tamaños poblacionales entre 0-4 y entre 45-50 no se reproducen.

Así, el evento climático conllevaría a una reducción abrupta en el crecimiento del pescado si la población tiene 28 peces o menos. < si los participantes preguntan por qué, se responde: los eventos climáticos puede reducir lluvias, aumentar temperatura y el riesgo de sequías. Esos eventos afectan la disponibilidad de nutrientes para los peces y la reproducción de los peces y otras especies en el ecosistema>

El asistente les entregará una tabla nueva mostrando la relación entre el tamaño poblacional y la reproducción después del evento climático, en caso de que ocurra *< entregar>.*

Nosotros tampoco sabemos si el evento climático ocurrirá. Ahora les cuento como determinamos si el evento climático ocurre en cada ronda:

Aquí tenemos dos urnas - una con 5 bolas verdes otra con 5 bolas rojas *<señalar>.* Las bolas de color rojo representan el evento climático, las bolas verde significan que no hay evento climático —la reproducción sigue igual. Ahora tomaremos 1 bolas verde y 1 bolas roja *< se mezclan en una urna no trasparente>.* [Nombre de asistente] tomara 8 bolas de las que quedaron en la urna, en donde mezclaremos las bolas verdes y rojas que quedaron. Sin embargo, ella lo hará con la urna cubierta, así que ninguno de nosotros sabremos la cantidad de bolas verdes y rojas que ella coja. *< sostenga la tela sobre ambas urnas de tal manera que nadie pueda ver (incluidos nosotros) cuales o cuantas bolas escoge>* . Ahora tenemos una urna con 10 bolas, 1 verde, 1 roja y 8 bolas cuyo color es incierto tanto para nosotros como para ustedes.

Al comienzo de cada ronda, antes de tomar sus decisiones individuales, de ahora en adelante [nombre de asistente] tomara una bola — pero ninguno de ustedes verá el color de la bola. — si ella toma una bola verde, nada pasa y todo sigue igual en la siguiente ronda. Pero si ella toma la bola roja, el evento climático ocurrirá. Eso significa que nosotros calcularemos el tamaño del recurso de acuerdo con la nueva relación entre el tamaño poblacional y su reproducción *<señalar nuevamente la tabla>.*

Como dije anteriormente, el evento tiene consecuencias duraderas. Esto significa que, una vez salga la bola roja, la nueva tabla de reproducción aplicará para el resto del juego. Nótese que sin importar el color de la bola, nosotros seguiremos sacando una bola al principio de cada ronda. Recuerde, el evento climático es impredecible y nosotros tampoco sabemos si ocurrirá o cuando.

Con esta nueva información, también re estableceremos el tamaño poblacional a 50 pescados <acá tache el número de peces que quedaron para la ultima ronda de la primera parte del juego y aclare que se inicia con 50 peces otra vez>, como en el comienzo del juego. El resto de las reglas se mantienen:

- Ustedes cuatro comparten una zona pesquera comunitaria
- En cada ronda usted tomará una decisión individual sobre cuantos peces quiere pescar. Usted puede escoger cuanto pescado haya disponible o no pescar (cero). Por cada pescado capturado usted gana 500 pesos. Usted también adivinará cuantos pescados quedaran para la siguiente faena.
- Sus decisiones afectan su bienestar individual pero también afectan el recurso de toda la comunidad.
- Siempre y cuando haya peces disponibles, esta parte del juego continuará y ustedes podrán seguir ganando plata. Si el recurso colapsa esta parte del juego termina.
- Nosotros no le diremos cuantas rondas va a jugar en esta parte del juego.
- La reproducción del pescado depende de cuanto pescado queda en la zona de pesca y de si el evento climático ocurrió o no *<señale la ilustración y recuérdeles las tablas>*
- Ustedes no pueden mostrarle lo que escriben en sus tarjetas de decisión a los otros pescadores
- Recuerde, ustedes pueden hablar durante todo el juego, Esto significa que usted puede hablar de lo que quiera incluso de cuánto pescado quiere sacar, por un máximo de 2 minutos entre cada ronda, después de esos dos minutos usted debe escribir su decisión.
-

La única diferencia, es que — puede que ocurra un evento climático en las siguientes rondas.

¿Tienen alguna pregunta?

Si no, continuemos jugando, El lugar de pesca común empieza teniendo 50 peces, ahora tienen dos minutos que pueden usar para discutir sus decisiones, después de este tiempo deberán escribir su decisión y nosotros recogeremos las carpetas

< Ejemplo en caso de que pregunten: Por ejemplo, si hay solo 10 peces disponibles pero la extracción colectiva es de 100 pescados (supongamos 30, 20, 30, 20), los pescados que quedaron serán divididos proporcionalmente a lo que usted quizo pescar. Así si su intención de pesca eran 30 peces de los 100 que como grupo intentaron extraer (30%), entonces usted recibirá el 30% de los peces que estaban disponibles en realidad, esto es 3 peces (30% de 10)>

Riesgo

[después de la ronda 6]

Ahora juguemos la segunda parte. En esta ronda, queremos informarle que el tamaño de la población para la siguiente ronda es […] <escriba el tamaño de la población de la última ronda de la primera parte del juego>También queremos informarle que un evento climático — con consecuencias negativas y duraderas para el recurso pesquero que ustedes manejan puede ocurrir.

Este tipo de evento climático es impredecible. Esto significa que usted no sabe si el evento va a ocurrir o cuando. Puede ocurrir en la siguiente ronda, en cuatro rondas, — o nunca…

En el caso de que el evento ocurra, — la reproducción del pescado se verá afectada, se reducirá por debajo del tamaño poblacional de 28 peces, como pueden ver aquí *<señale la nueva ilustración con dinámica de umbral>* la reproducción será únicamente de un pez para tamaños poblacionales entre 2 y 27 pescados. Para los demás tamaños poblacionales la reproducción será la misma, entre 28-34 serán 10 pescados, entre 35-45 serán 5 pescados, como pueden ver aquí <señalar>. Los tamaños poblacionales entre 0-4 y entre 45-50 no se reproducen.

Así, el evento climático conllevaría a una reducción abrupta en el crecimiento del pescado si la población tiene 28 peces o menos. < si los participantes preguntan por que, se responde: los eventos climáticos puede reducir lluvias, aumentar temperatura y el riesgo de sequías. Esos eventos afectan la disponibilidad de nutrientes para los peces y la reproducción de los peces y otras especies en el ecosistema>

El asistente les entregará una tabla nueva mostrando la relación entre el tamaño poblacional y la reproducción después del evento climático, en caso de que ocurra *< entregar>.*

Nosotros tampoco sabemos si el evento climático ocurrirá. Ahora les cuento como determinamos si el evento climático ocurre en cada ronda:

Aquí tenemos dos urnas - una con 5 bolas verdes otra con 5 bolas rojas *<señalar>.* Las bolas de color rojo representan el evento climático, las bolas verde significan que no hay evento climático —la reproducción sigue igual. [Nombre de asistente] tomara las 10 bolas (5 verdes y 5 rojas) y las mezclará en la urna. Al comienzo de cada ronda, antes de tomar sus decisiones individuales, de ahora en adelante [nombre de asistente] tomara una bola — pero ninguno de ustedes verá el color de la bola. — si ella toma una bola verde, nada pasa y todo sigue igual en la siguiente ronda. Pero si ella toma la bola roja, el evento climático ocurrirá. Eso significa que nosotros calcularemos el tamaño del recurso de acuerdo con la nueva relación entre el tamaño poblacional y su reproducción *<señalar nuevamente la tabla>.*

Como dije anteriormente, el evento tiene consecuencias duraderas. Esto significa que, una vez salga la bola roja, la nueva tabla de reproducción aplicará para el resto del juego. Nótese que sin importar el color de la bola, nosotros seguiremos sacando una bola al principio de cada ronda. Recuerde, el evento climático es impredecible y nosotros tampoco sabemos cuando ocurrirá, pero ambos sabemos que la probabilidad es 50-50.

Con esta nueva información, también re estableceremos el tamaño poblacional a 50 pescados <acá tache el número de peces que quedaron para la ultima ronda de la primera parte del juego y aclare que se inicia con 50 peces otra vez>, como en el comienzo del juego. El resto de las reglas se mantienen:

- Ustedes cuatro comparten una zona pesquera comunitaria
- En cada ronda usted tomará una decisión individual sobre cuantos peces quiere pescar. Usted puede escoger cuanto pescado haya disponible o no pescar (cero). Por cada pescado capturado usted gana 500 pesos. Usted también adivinará cuantos pescados quedaran para la siguiente faena.
- Sus decisiones afectan su bienestar individual pero también afectan el recurso de toda la comunidad.
- Siempre y cuando haya peces disponibles, esta parte del juego continuará y ustedes podrán seguir ganando plata. Si el recurso colapsa esta parte del juego termina.
- Nosotros no le diremos cuantas rondas va a jugar en esta parte del juego.

- La reproducción del pescado depende de cuanto pescado queda en la zona de pesca y de si el evento climático ocurrió o no *<señale la ilustración y recuérdeles las tablas>*
- Ustedes no pueden mostrarle lo que escriben en sus tarjetas de decisión a los otros pescadores
- Recuerde, ustedes pueden hablar durante todo el juego, Esto significa que usted puede hablar de lo que quiera incluso de cuánto pescado quiere sacar, por un máximo de 2 minutos entre cada ronda, después de esos dos minutos usted debe escribir su decisión.
-

La única diferencia, es que — puede que ocurra un evento climático en las siguientes rondas.

¿Tienen alguna pregunta?

Si no, continuemos jugando, El lugar de pesca común empieza teniendo 50 peces, ahora tienen dos minutos que pueden usar para discutir sus decisiones, después de este tiempo deberán escribir su decisión y nosotros recogeremos las carpetas

< Ejemplo en caso de que pregunten: Por ejemplo, si hay solo 10 peces disponibles pero la extracción colectiva es de 100 pescados (supongamos 30, 20, 30, 20), los pescados que quedaron serán divididos proporcionalmente a lo que usted quizo pescar. Así si su intención de pesca eran 30 peces de los 100 que como grupo intentaron extraer (30%), entonces usted recibirá el 30% de los peces que estaban disponibles en realidad, esto es 3 peces (30% de 10)>

Efecto de umbral

[después de la ronda 6]

Ahora juguemos la segunda parte. En esta ronda, queremos informarle que el tamaño de la población para la siguiente ronda es […]<escriba el tamaño de la población de la última ronda de la primera parte del juego> También queremos informarle que un evento climático — con consecuencias negativas y duraderas para el recurso pesquero que ustedes manejan ocurrirá.

El evento climático afectará la reproducción del pescado, se reducirá por debajo del tamaño poblacional de 28 peces, como pueden ver aquí *<señale la nueva ilustración con dinámica de umbral>* la reproducción será únicamente de un pez para tamaños poblacionales entre 2 y 27 pescados. Para los demás tamaños poblacionales la reproducción será la misma, entre 28-34 serán 10 pescados, entre 35-45 serán 5 pescados, como pueden ver aquí <señalar>. Los tamaños poblacionales entre 0-4 y entre 45-50 no se reproducen.

Así, el evento climático conlleva a una reducción abrupta en el crecimiento del pescado si la población tiene 28 peces o menos. < si los participantes preguntan por que, se responde: los eventos climáticos puede reducir lluvias, aumentar temperatura y el riesgo de sequías. Esos eventos afectan la disponibilidad de nutrientes para los peces y la reproducción de los peces y otras especies en el ecosistema>

El asistente les entregará una tabla nueva mostrando la relación entre el tamaño poblacional y la reproducción después del evento climático *< entregar>.* A partir de esta ronda y por el resto del juego el evento climático ocurrirá y afectará la reproducción del recurso.

Con esta nueva información, también re estableceremos el tamaño poblacional a 50 pescados <acá tache el número de peces que quedaron para la ultima ronda de la primera parte del juego y aclare que se inicia con 50 peces otra vez> pescados, como en el comienzo del juego. El resto de las reglas se mantienen:

- Ustedes cuatro comparten una zona pesquera comunitaria
- En cada ronda usted tomará una decisión individual sobre cuantos peces quiere pescar. Usted puede escoger cuanto pescado haya disponible o no pescar (cero). Por cada pescado capturado usted gana 500 pesos. Usted también adivinará cuantos pescados quedaran para la siguiente faena.
- Sus decisiones afectan su bienestar individual pero también afectan el recurso de toda la comunidad.
- Siempre y cuando haya peces disponibles, esta parte del juego continuará y ustedes podrán seguir ganando plata. Si el recurso colapsa esta parte del juego termina.
- Nosotros no le diremos cuantas rondas va a jugar en esta parte del juego.
- La reproducción del pescado depende de cuanto pescado queda en la zona de pesca y de si el evento climático ocurrió o no *<señale la ilustración y recuérdeles las tablas>*
- Ustedes no pueden mostrarle lo que escriben en sus tarjetas de decisión a los otros pescadores
- Recuerde, ustedes pueden hablar durante todo el juego, Esto significa que usted puede hablar de lo que quiera incluso de cuánto pescado quiere sacar, por un máximo de 2 minutos entre cada ronda, después de esos dos minutos usted debe escribir su decisión.

La única diferencia, es que — un evento climático ocurrió y de ahora en adelante la recuperación de recurso es menor si la población es menor que 28 pescados

¿Alguna pregunta?
Si no, continuemos jugando, El lugar de pesca común empieza teniendo 50 peces, ahora tienen dos minutos que pueden usar para discutir sus decisiones, después de este tiempo deberán escribir su decisión y nosotros recogeremos las carpetas

< Ejemplo en caso de que pregunten: Por ejemplo, si hay solo 10 peces disponibles pero la extracción colectiva es de 100 pescados (supongamos 30, 20, 30, 20), los pescados que quedaron serán divididos proporcionalmente a lo que usted quizo pescar. Así si su intención de pesca eran 30 peces de los 100 que como grupo intentaron extraer (30%), entonces usted recibirá el 30% de los peces que estaban disponibles en realidad, esto es 3 peces (30% de 10)>

Línea Base

[después de la ronda 6]

Ahora juguemos la segunda parte. En esta ronda, queremos informarle que el tamaño de la población para la siguiente ronda es […]<escriba el tamaño de la población de la última ronda de la primera parte del juego> También queremos informarle que este año ha sido muy bueno y el recurso se recuperó mas de lo normal. El tamaño del cardumen es de 50 pescados <acá tache el número de peces que quedaron para la ultima ronda de la primera parte del juego y aclare que se inicia con 50 peces otra vez>, como en el comienzo del juego. Y las reglas del juego son las mismas:

- Ustedes cuatro comparten una zona pesquera comunitaria
- En cada ronda usted tomará una decisión individual sobre cuantos peces quiere pescar. Usted puede escoger cuanto pescado haya disponible o no pescar (cero). Por cada pescado capturado usted gana 500 pesos. Usted también adivinará cuantos pescados quedaran para la siguiente faena.
- Sus decisiones afectan su bienestar individual pero también afectan el recurso de toda la comunidad.
- Siempre y cuando haya peces disponibles, esta parte del juego continuará y ustedes podrán seguir ganando plata. Si el recurso colapsa esta parte del juego termina.
- Nosotros no le diremos cuantas rondas va a jugar en esta parte del juego.
- La reproducción del pescado depende de cuanto pescado queda en la zona de pesca y de si el evento climático ocurrió o no *<señale la ilustración y recuérdeles las tablas>*
- Ustedes no pueden mostrarle lo que escriben en sus tarjetas de decisión a los otros pescadores
- Recuerde, ustedes pueden hablar durante todo el juego, Esto significa que usted puede hablar de lo que quiera incluso de cuánto pescado quiere sacar, por un máximo de 2 minutos entre cada ronda, después de esos dos minutos usted debe escribir su decisión.

La única diferencia, es que — un evento positivo hizo que el cardumen creciera mas de lo normal y comenzaremos con 50 pescados nuevamente.

¿Alguna pregunta?

Si no, continuemos jugando, El lugar de pesca común empieza teniendo 50 peces, ahora tienen dos minutos que pueden usar para discutir sus decisiones, después de este tiempo deberán escribir su decisión y nosotros recogeremos las carpetas

< Ejemplo en caso de que pregunten: Por ejemplo, si hay solo 10 peces disponibles pero la extracción colectiva es de 100 pescados (supongamos 30, 20, 30, 20), los pescados que quedaron serán divididos proporcionalmente a lo que usted quizo pescar. Así si su intención de pesca eran 30 peces de los 100 que como grupo intentaron extraer (30%), entonces usted recibirá el 30% de los peces que estaban disponibles en realidad, esto es 3 peces (30% de 10)>

< Este juego se hace cuando el termina el juego de la pesca. El líder del experimento introduce el juego y el asistente circula las hojas de decisión. Cada pescador toma dos decisiones individuales. Al final mediante una lotería se escoge a una persona a quien se le pagara basado en una sola decisión. Todavía me falta editar esta parte pero no debe tomar mas de 5min después del juego de la pesca. >

Instrucciones juego de riesgo y ambigüedad

En esta actividad usted tomará 2 decisiones. Al final si esta actividad sale al azar, escogeremos un jugador y una de las decisiones también al azar, para pagarle este ejercicio. Usted podrá ganar la cantidad de dinero de estas decisiones, por lo que es muy importante que piense cada decisión muy bien.
-usar el "Afiche Actividad 3 ejemplos"-
En cada decisión tendrá al frente seis sobres diferentes. Cada círculo representa una de los seis sobres. Dentro de cada sobre hay 10 fichas. Cada ficha representa la cantidad de dinero que Usted se ganaría si saca esa ficha del sobre. Su decisión consiste en escoger un solo sobre de los seis y de ese sobre únicamente podrá sacar al final una ficha para saber cuánto se gana.

Como le dijimos, dentro de cada sobre hay 10 fichas. Solamente uno de los seis sobres tiene fichas que representan la misma cantidad de dinero. En los demás sobres la mitad de las fichas representa un pago más bajo y la otra mitad un pago más alto. *(Coordinador: mostrar sobre $4000/$31000 de ejemplo)*. Los valores altos y bajos son diferentes en cada uno de los seis sobres, por eso debe considerar muy bien cuál sobre prefiere jugar antes de sacar una ficha de ese sobre. *(Coordinador: comparar $4000/$31000 con $7000/$25000 para mostrar la diferencia).*

<Para el siguiente juego por favor cambie el orden en la mañana haga primero riesgo y después ambigüedad y el la tarde haga primero ambigüedad y después riesgo)

Riesgo (Decisión 1 en la mañana- Decisión 2 en la tarde)

Coordinador: Tome el sobre $10000/$19000 del primer grupo de 6 y demuestre el mecanismo de cómo funcionará el juego.

Miremos con un ejemplo como funcionará esta actividad. Suponga que de los seis sobres que tiene al frente Usted escoge el sobre con 5 fichas de $10000 y 5 fichas de $19000. Para marcar esa opción usted pone una X en el sobre. Vamos a construir ese sobre. Aquí tenemos un sobre vacío en el cual depositamos las 5 fichas que representan el valor bajo de esta opción. Es decir, $10000. Y ahora depositamos las 5 fichas que representan el valor alto $19000. Suponga que al final se paga esta actividad y esta decisión. Saque por favor una ficha del sobre para definir su pago. Como sacó la ficha ____ sus ganancias en esta parte serán de $______.

Si debe hacer más ejemplos tome el sobre $2000/$36000 del primer grupo de 6. En caso de necesitar otro ejemplo utilice el sobre $13000/$13000

¿Hay alguna pregunta?

Por favor abra el librillo y tome su decisión en esa página.
Para escoger un sobre, márquelo con una X. Por favor marque solo un sobre.
Una vez marque un sobre pase a la página siguiente.

Ambigüedad (Decisión 1 en la tarde - Decisión 2 en la mañana)

Ahora vamos a explicar en que consiste la segunda decisión. Después de hacerlo con el primer grupo de seis sobres Usted volverá a hacerlo con otros seis sobres que tienen los mismos valores para las fichas, pero Usted no sabrá con exactitud cuántas fichas hay de cada valor. Cuatro fichas podrán ser de valor bajo o alto pero esto usted no lo sabrá al escoger el sobre. Solo sabrá que hay 3 fichas del valor bajo y 3 fichas del valor alto.
Coordinador: Tome sobre $10000/$19000 del segundo grupo de 6 y demuestre el mecanismo de cómo funcionará el juego.

Miremos con un ejemplo como funcionará esta segunda decisión. Suponga que de los seis sobres que tiene al frente Usted escoge el sobre con 3 fichas de $10000, 3 fichas de $19000 y 4 fichas cuyo valor usted no lo sabe. Esas cuatro fichas pueden ser o del valor alto del sobre o del valor bajo *(marque con una X la opción)*. Vamos a construir ese sobre. Primero, para escoger las cuatro fichas cuyo valor no sabemos metemos en otro sobre vacío 4 fichas del valor alto y 4 fichas del valor bajo. Revolvemos el sobre y sacamos 4 fichas sin mirar. Estas 4 fichas pueden ser de valor alto o bajo, no se sabe, y las depositamos en el sobre vacío que representa esta opción. Igualmente, depositamos las 3 fichas que representan el valor bajo de esta opción. Es decir, $10000. Y ahora depositamos las 3 fichas que representan el valor alto $19000. Suponga que al final se paga esta actividad y esta decisión. Saque por favor una ficha del sobre para definir su pago. Como sacó la ficha ____ sus ganancias en esta parte serán de $______.

Si debe hacer más ejemplos tome el sobre $2000/$36000 del segundo grupo de 6. En caso de necesitar otro ejemplo utilice el sobre $13000/$13000

¿Hay alguna pregunta?

Por favor tome su decisión.
Para escoger un sobre, márquelo con una X. Por favor marque solo un sobre.
