# Supplementary material for: Cooperation in the face of thresholds, risk, and uncertainty: Experimental evidence in fisher communities from Colombia
Source: PLoS One. 2020 Dec 28;15(12):e0242363. doi: 10.1371/journal.pone.0242363 (PMC7769437; doi:10.1371/journal.pone.0242363)
Supplement: Attachment — Submitted filename: Review - PONE-D-20-19709.pdf [file pone.0242363.s003.pdf]

Review: Cooperation in the face of thresholds, risk, and uncertainty

This manuscript presents experimental evidence on the cooperative behaviours of individuals when faced to the risk of environmental crisis. Its main contribution is to provide insights on the behaviours of individuals used to manage resources, in contrast to previous work mostly focusing on WEIRD societies.

On the positive side, the manuscript provides a valuable contribution because (i) experimental evidence is always relevant (and this is particularly true in the field of evolution of cooperation dominated by theoretical work), and (ii) because the results of this study challenge previous conclusions mostly based on WEIRD societies. The introduction and the discussion are well written and referenced. The goal of the manuscript is well motivated. Previous work on the topic are cited and the authors do a good work at contextualising their study, either to motivate the study as in the introduction, or to connect their results in the discussion. The experimental design is solid. The authors show an expertise in statistics and data analysis, even if the complexity of the statistical analysis can sometimes limit the understanding of a reader.

The negative point is mainly the results section and the analysis (besides the statistical analysis) presented. First, some choices in the experimental design and the analysis lack justification (or alternatively, discussion of the consequences of these choices). For instance, the index created to measure cooperation appears arbitrary (arbitrary value in some cases, replenishment rate not taken in account, …). The rationale behind the effects of the climate event is not clear. Authors sometimes use median and sometimes use the mean. Altogether, this can lead readers to doubt the robustness of the conclusion of the study. Second, the results section strongly needs rewriting. On the one hand, a large part of the results section is not result but the description of the method. On the other hand, the space dedicated to the actual results and analysis is limited with for instance, the main result on cooperation appears to be missing. Moreover, the plots are too complex and often not clear. Ultimately, this results in readers having to trust the conclusions and interpretations of the authors rather than reaching the same conclusions than the authors throughout the analysis.

To conclude, I favour publication, but at the condition of rewriting the results section.

You can find below the detailed list of comments:

Title

- Change the title to make it clear that the paper presents experimental evidence. For instance, add “Experimental evidence on […]” or “[…] in fishers communities from Colombia”.

Introduction

- Some details at the end of the introduction should be moved to the next section. Authors should reconsider having a method section between introduction and results rather than at the end.
- The authors might be interested by theoretical work by Francisco C. Santos on the topic of cooperation with risk (for instance, https://www.pnas.org/content/pnas/108/26/10421.full.pdf) .

Method

- A figure that explains the replenishment rate would be helpful. For instance, the figure could be a line representing the population size of the fish stock, divided in sections for the different replenishment rate.
- What are the effects of the diminishing returns? It could be argued that individuals taking a lot of fishes are actually cooperating because they reduce the population size down to the maximal productivity. If it does not matter e.g. it rarely happens, add a sentence stating it.
- The climate event (i) reduces replenishment rate of low population size and (ii) reduces the interval of population size where the replenishment rate is optimal but does not affect the replenishment rate of this interval. What is the rationale behind this choice? For instance, why did not the authors consider that climate event simply reduces replenishment rate for any population size? As far as I know, this differs from most of theoretical work so how does this choice affects the results presented, and the comparison with theoretical work?
- Why does the fish stock is restored at turn 7? Did fishers know about this? Can it affect the results?
- L266 "There was no reproduction …" -> Move this sentence up, where you explain the different replenishment rates.
- L259: "The event was meant to reduce …" -> "The event reduces …"
- L297: remove the "than expected" and "initially planned".
- Did the fishers know the details about the different replenishment rates?
- L299: Add a reference for similar surveys in the literature to motivate the choice of the survey (if it exists).

Analysis

- Why does the authors use median cooperation but mean extraction?
- Check if this notation is "p = 0.1:0.9" is commonly used. Change to $0.1 < p < 0.9$ instead?
- L88 – L95. This should be in the method section.
- L99 -L128: This should be in the method section
- L99-105: There is no need to discuss the different definitions of cooperation in the literature. One sentence explaining cooperation and how it is defined in this study is enough.
- The measure of cooperation used lacks justification. This can lead readers to doubt of the results on cooperation, which is problematic because this is the main result. I understand the difficulty to create an index that take in account both the number of fish taken and the situation of the fish stock (above or below the threshold). I would advise to either split the index in two, with one index describing if individuals take fish when the stock is below the threshold, and one index describing the amount of fishes taken when above the threshold. Alternatively, the author needs to better justify the index built.
    - Provide the rationale behind the choice made to build the index:
        - For instance, "We consider that cooperation is represented by individuals maintaining the fish stock in its most productive/sustainable state. Thus, cooperation happens when (i) individuals take a number of fishes that maintain the fish stock above the threshold, or (ii) take no fishes when the fish stock is below the threshold"
        - Using biological terms rather than mathematical terms helps readers understand the choice made to built the index. For instance: "To avoid division by zero or negative values, if the denominator is < 1 and x_i,t = 0

cooperation is set to C = 1 (212/4096 observations), […]" can be replaced by "When the fish stock is below the threshold (denominator < 1) and fishers do not take any fish ($x_{i,t}$ = 0), we consider that they cooperate C = 1".
    - The authors consider that fishers taking fish while the stock is below the threshold results in a cooperation value of 1.5. This value is arbitrary so what does happen when a different value is used?
    - It is confusing that a higher value of the cooperation index means lower cooperation. Change the index to (1 – $C_{i,t}$) or change the name of the index.
    - Why does the replenishment rate is not taken in account in the cooperation index?
- L120-123. It is not clear why the authors justify the choice of describing coordination this way. If the authors use the average cooperation across different rounds, deviation from cooperation for a single round should not be a problem. I would remove this part. Simply introduce coordination in a way to measure coordination.
- L128: Present the results of the effects of treatments on cooperation and coordination before starting to explain these effects. So far, the results section seems to lack the main result (no effect of treatments on cooperation).
- L141: Not clear and this choice looks arbitrary. Add justification or reference.
- L149. Split the sentence for clarity. First part is about all treatments having an effect and second part is about a single treatment having an effect.
- L157: But do individuals that reach agreement are explained by socio-economic effects?
- The results section ends without having the main result (no effect of treatment on cooperation) being clearly presented. It appears that the result section starts by describing the effect of treatments on the number/proportion of fishes taken and then jumps directly to how these effects can be explained (socio-economic factors, coordination and agreement).

Figures

- Figure 1:
    - What is before and after? I do not find explanations in the text. Does that mean that the cooperation presented is averaged on the rounds before and after the round 7?
    - I would advise to start the caption by a sentence presenting the plots, and then have a sentence describing more formally the analysis. For instance, "Effect of treatment (risk, threshold, uncertainty) on the individual extraction (top), proportion of stock (middle) and cooperation (bottom). The effects of the treatment are tested using …"
    - Replace "counterfactual" by "baseline" in the line type (or explain in the caption).
- Figure 2 is not clear at all.
    - First, it can be improved in term of appearance, e.g. the quality is low, the number of different plots is too high, the size of the plots change.
    - Second, a plot needs to support one or two conclusions rather than providing an exhaustive presentation of the results (this goes into supplementary materials). Split this figure in different figures.
- Figure 3
    - If possible, colour the points as a function of their p-values, in the same way than Figure 1.

Discussion

- L175: First sentence is not clear.

- L175: “Fishers under uncertain thresholds maintained higher levels of cooperation than when the risk of thresholds was known, but risk had a stronger effect at reducing individual fishing effort than uncertainty.” L207: “However, cooperation as measured in our study was not affected by our treatments.”. These two statements seem to contradict each other.
- L180: The authors state that uncertainty increase cooperation, but I thought that uncertainty did not affect the level of cooperation.
- L211: The author could cite Elinor Ostrom, e.g. Governing the commons (1990).

Typo

- A comma is often missing “,”: L316: “For risk the chances […]”, L360: “As response variables we used […]”.
- Fig 1 caption: “contorl”.
- L184 “effots”
- Fig2 caption: Add “Figure” before “A) and B)”
